# Impact of Lu-Substitution in Yb_14–*x*_Lu_*x*_ZnSb_11_: Thermoelectric Properties and Oxidation Studies

**DOI:** 10.1021/acsaem.3c01756

**Published:** 2023-10-04

**Authors:** Andrew P. Justl, Logan D. Winston, Sabah K. Bux, Susan M. Kauzlarich

**Affiliations:** † Department of Chemistry, University of California, One Shields Ave, Davis, California 95616, United States; ‡ Thermal Energy Conversion Technologies Group, Jet Propulsion Laboratory, California Institute of Technology, 4800 Oak Grove Drive, MS 277-207, Pasadena, California 91109, United States

**Keywords:** zintl, 14-1-11, oxidation kinetics, thermoelectrics, Ca_14_AlSb_11_, Lu doping, Yb_14_ZnSb_11_, high temperature materials, p-type semiconductor

## Abstract

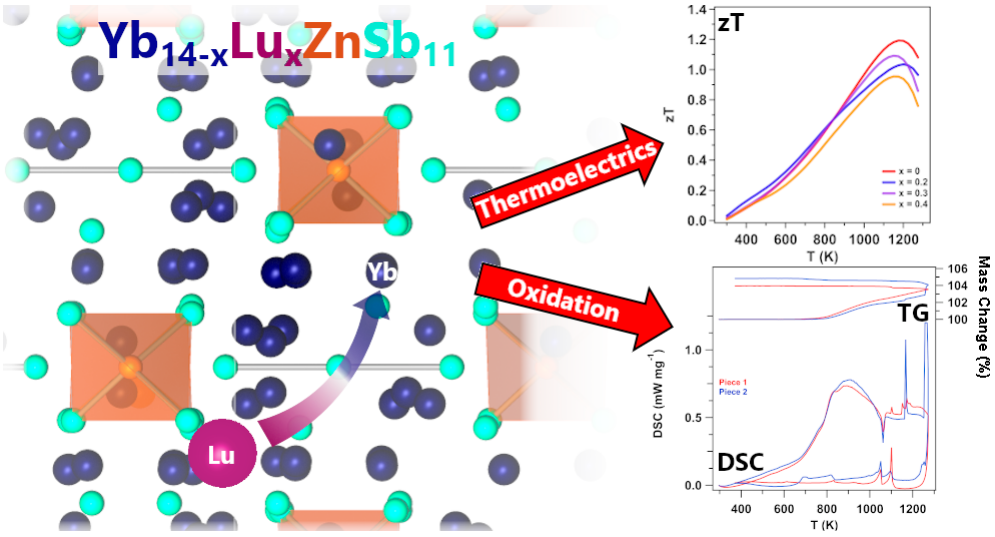

Yb_14_ZnSb_11_ is one of the newest additions to the high-performance Yb_14_MSb_11_ (M = Mn, Mg, and Zn) family of p-type high-temperature thermoelectric materials and shows promise for forming passivating oxide coatings. Work on the oxidation of rare earth (RE)-substituted Yb_14–*x*_RE_*x*_MnSb_11_ single crystals suggested that substituting late RE elements may form more stable passivation oxide coatings. Yb_14–*x*_Lu_*x*_ZnSb_11_ (*x* = 0.1, 0.2, 0.3, 0.4, 0.5, and 0.7) samples were synthesized, and Lu-substitution’s effects on thermoelectric and oxidation properties are investigated. The solubility of Lu within the system was found to be quite low with *x*_max_ ∼ 0.3; samples with *x* > 0.3 contained impurities of LuSb. Goldsmid–Sharp band gap estimations show that introducing Lu reduces the apparent band gap. Because of this, the Lu-substituted samples show a reduction in the maximum Seebeck coefficient, decreasing the high-temperature *zT*. This contrasts with the impact of Lu^3+^ substitution in Yb_14_MnSb_11_, where the addition of Lu^3+^ for Yb^2+^ results in increases in both resistivity and the Seebeck coefficient. Oxidation of the *x* = 0.3 solid solution was studied by thermogravimetric– differential scanning calorimetry , powder X-ray diffraction, scanning electron microscopy–energy-dispersive spectroscopy, and optical images. The samples show no mass gain before 785 K, and ensuing oxidation reactions are proposed. At the highest temperatures, significant amounts of Yb_14–*x*_Lu_*x*_ZnSb_11_ remained beneath an oxide coating, suggesting that passivation may be achievable in oxygen environments.

## Introduction

Thermoelectric generators harness heat to produce electricity by utilizing the Seebeck effect, which describes the movement of electronic carriers in metals and semiconductors when a thermal gradient is applied. P- and n-type thermoelectric materials are oriented along the thermal gradient in parallel and electrically in series to harness this heat. This configuration allows electronic carriers to diffuse from hot to cold and move in opposite directions within the electronic circuit, leading to a useable current. The efficiency at which any material can convert that thermal gradient into electricity at a given temperature is judged by the unitless thermoelectric figure of merit, *zT* = *S*^2^*T*/ρκ. In this equation, *S* is the Seebeck coefficient or voltage created per degree of thermal gradient, *T* is the absolute temperature, rho (ρ) is the electrical resistivity, and kappa (κ) is the thermal conductivity.

These devices are attractive for applications where high durability and little to no maintenance requirements are a high priority because they have no moving parts and a high level of redundancy due to the many iterations of each thermoelectric leg. One application that exemplifies this is using radioisotope thermoelectric generators (RTGs) for power on deep space missions such as Voyagers I & II, Cassini, Curiosity, and Perseverance.^1,2^ RTGs use the decay of radioisotope pucks as a heat source, creating a self-sufficient system that can provide long-term reliable power to the mission. While these systems are robust and proven, oxidation of the legs can harm long-term performance. Small amounts of oxygen and moisture are difficult to entirely remove from insulation, and the enclosure of the generator and many thermoelectric materials are prone to oxidation at operating temperatures. This oxidation may negatively impact the performance and stability of the thermoelectric legs and in turn the generator.^3,4^

**Figure 1:**
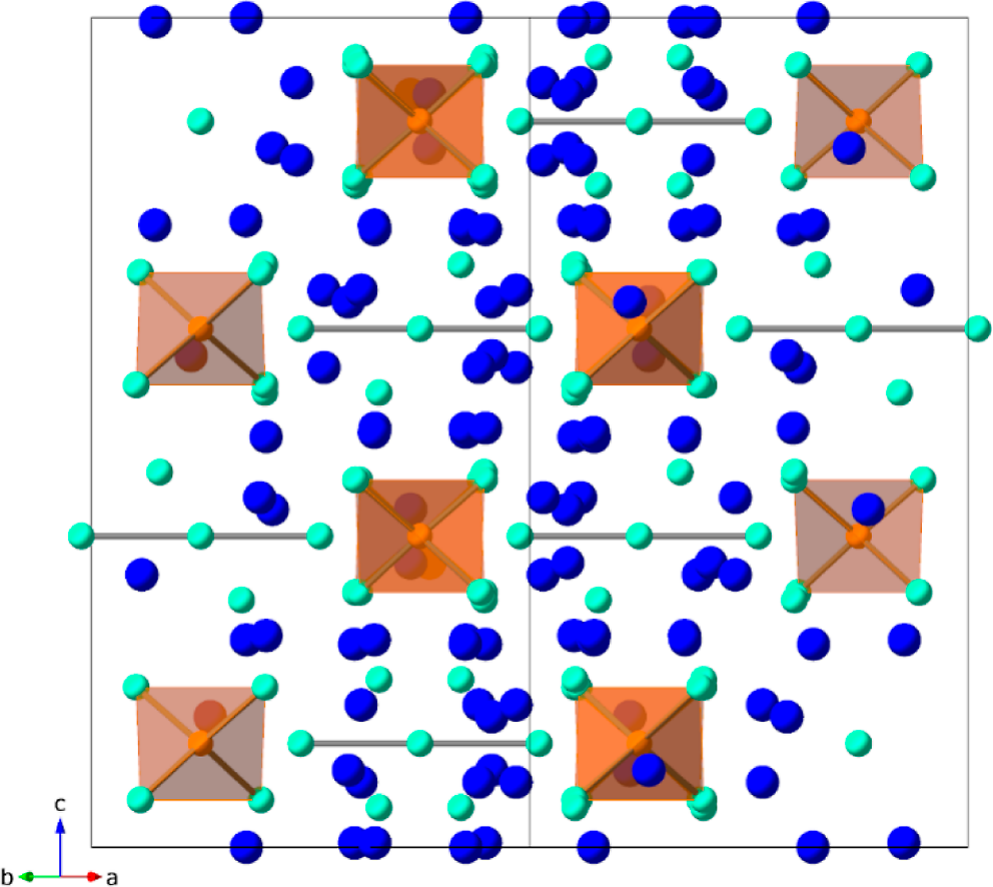
Unit cell of Yb_14_ZnSb_11_ is shown down the [110] plane. Here, Yb is indicated in dark blue, Sb in teal, and Zn in orange tetrahedra.

Recently, it was shown that Yb_14_ZnSb_11_ exhibits promising high-temperature (>1000 K) thermoelectric properties when made in high purity.^5^ The structure of Yb_14_ZnSb_11_ (Figure 1) can be described using the Zintl formulizm as consisting of ZnSb_4_^10–^ + Sb_3_^7–^ + 4Sb^3–^ + 13Yb^2+^ + 1Yb^3+^. Because the crystal structure does not have a site available for the single Yb^3+^, intermediate valency of Yb exists, and an average valency of 2.07 for all 14 Yb atoms provides the precise accounting.^6^ Studies on the magnetism within this system have shown that Yb_14_ZnSb_11_ exhibits a fluxional valency with Yb^2+/3+^, in which the amount of Yb^3+^ within the system decreases with temperatures.^7^ This deviation from the ideal zintl formulism in its lowest energy state leads to the system being electron deficient and heavily doped p-type (∼1 × 10^21^ h^+^ cm^–3^).^5^ The large complex unit cell filled with heavy atoms lends to ultralow thermal conductivities, while degeneracy within the band structure leads to high Seebeck coefficients while maintaining low electrical resistivities all of which are ideal for high thermoelectric performance.^5^

The oxidation process of Yb_14_ZnSb_11_ as a function of temperature was investigated and compared to the other high-performance analogues, Yb_14_MnSb_11_ and Yb_14_MgSb_11_, and showed the most promising passivating oxide shell formation.^8^ In addition, Yb_14_ZnSb_11_ does not appear to undergo a peritectic with increasing temperature, as observed in the large-scale pellet oxidation of the Mn and Mg analogues. Thermochemical studies on single crystals of Yb_14–*x*_RE_*x*_MnSb_11_ (RE = La–Nd and Sm–Lu) suggested that the substitution of Yb for the late rare earth (RE) elements led to improved oxidation resistance as they quickly formed a more stable oxide shell.^9^ Previously, Lu-substitution in polycrystalline Yb_14_MnSb_11_ showed some improvements in oxidation resistance; however, the YbMnSb_2_–YbSb_2_ eutectic lead to molten material forming underneath and breaking through the oxide shell at high temperatures.^10^ In this work, Yb_14_ZnSb_11_ is substituted with trivalent RE, Lu. Polycrystalline samples of Yb_14–*x*_Lu_*x*_ZnSb_11_ (*x* = 0.1–0.7) are synthesized from Lu, YbH_2_, Yb_4_Sb_3_, and ZnSb, and the effects of trivalent RE substitution on the thermoelectric and oxidation properties are investigated.

## Experimental Section

### Synthesis of Binary Precursors

#### ZnSb

Reaction was performed with 5 g of total mass. Stoichiometric amounts of Zn pieces (Columbus Chemical Industries, 99.98%) and Sb shot (5N Plus, 99.999%) were added to a 65 cm^3^ stainless-steel grinding vial with two 12.7 mm diameter balls (SPEX) inside an Ar filled glovebox (<0.5 ppm of O_2_). The vial was closed and sealed in Mylar inside the glovebox before being milled with an SPEX high energy mill for four rounds of 1 h with 15 min pauses in between each round to moderate the temperature. After milling, the gray powder can be indexed as ZnSb (ICSD 55403) with a small amount of unreacted Zn and Sb. To complete the reaction, the powder was removed from the grinding vial inside a glovebox and transferred to a Nb tube, which was welded shut on both ends under an inert atmosphere. The sealed Nb tube was further jacketed in fused silica under a vacuum to prevent oxidation of the Nb tube upon heating. The reaction mixture was heated to 450 °C and held for 12 h. The resultant gray powder was identified as pure phase ZnSb using powder X-ray diffraction (PXRD).

#### Yb_4_Sb_3_

The reaction was done in a 10 g total mass. Stoichiometric amounts of Yb cuttings (<3 × 3 × 3 mm) from a larger ingot (Stanford Materials, 99.99%) and Sb shot (5N Plus, 99.999%) were added to a 55 cm^3^ tungsten carbide grinding vial with two 11.2 mm diameter tungsten carbide balls (SPEX) inside an Ar filled glovebox (<0.5 ppm of O_2_). The higher density/energy of the tungsten carbide balls helps when milling large batches and difficult materials. The reaction was sealed in Mylar, removed from the glovebox, and milled for three rounds of 30 min. In between each round, the walls and lids of the reaction vial were scraped using a chisel inside the glovebox to remove the cold welded Yb and stuck-on material. This is important for improving reaction homogeneity, stoichiometry, and, in turn, purity. After milling, the black powder was removed from the vial by using a chisel inside the glovebox. The powder was transferred to a Nb tube, which was then welded shut under Ar and further jacketed in a fused silica tube under vacuum. The reaction mixture was heated to 850 °C and kept at that temperature for 12 h. Because of small amounts of Yb loss to the grinding vial, the resultant black powder can be identified by PXRD as Yb_4_Sb_3_ with a small amount (<15 wt %) of the Yb deficient, adjacent phase, Yb_11_Sb_10_. Because this can be described as a single point within the Yb–Sb phase diagram, the powder was treated as homogeneous. If other phases (Yb_2_O_3_ or other Yb–Sb binaries) are present, the powder was not used, as this suggests that there is an uneven distribution of elements or unwanted oxides. To compensate for the Yb_11_Sb_10_ impurity, Rietveld refinement was performed on the PXRD patterns to obtain relative mass fractions of Yb_4_Sb_3_ and Yb_11_Sb_10_, which were then converted to mole fraction (chi, χ) which was used to calculate the reaction to make Yb_14–*x*_Lu_*x*_ZnSb_11_, as described previously.^11^

Using the results from a Rietveld refinement of the Yb_4_Sb_3_ powder, the final reaction to form Yb_14–*x*_Lu_*x*_ZnSb_11_ can be solved. Similar to what was employed to make Yb_14_ZnSb_11_, a three variable, three equation system of equations can be used to solve for the coefficients in eq 1 below.^11^
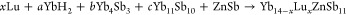
1
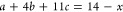
2
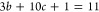
3
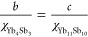
4

Equation 1: the chemical reaction formed Yb_14–*x*_Lu_*x*_MnSb_11_ from Lu, YbH_2_, and Yb_4_Sb_3_ with Yb_11_Sb_10_ impurities. Equations 2–4: the three equations used to solve for the variables are given in eq 1. The content of Yb and Lu in Yb_14–*x*_Lu_*x*_ZnSb_11_ is determined by eq 2, where the desired Lu content (*x*) needs to be predetermined. The Sb content is fixed at 11 by eq 3. Equation 4 uses the relationship *n*_total_ × χ_A_ = *n*_A_ to define the equality between the mole fractions (χ, chi) of Yb_4_Sb_3_ and Yb_11_Sb_10_ from Rietveld refinement, which are denoted by their respective subscripts.

Using eqs 2–4 and the results from the Rietveld refinement of the Yb_4_Sb_3_ powder, the variables within eq 1 can be solved to get the desired balanced reaction. Beginning with eq 4, both mole fractions are known values from the refinement, so that the equation can be used in a substitution for either variable within eq 3. Once one variable is solved, the other is easily obtained. Equation 2 can then be solved. Once all variables are obtained, they can be inserted into eq 1 to obtain the balanced reaction to form Yb_14–*x*_Lu_*x*_ZnSb_11_ from Lu, YbH_2_, ZnSb, and Yb_4_Sb_3_ (with or without Yb_11_Sb_10_ impurities). Because compositions of the precursor vary, it is important to solve this for each individual batch of Yb_4_Sb_3_.

### Synthesis of Yb_14–*x*_Lu_*x*_ZnSb_11_

Reactions were done in 5 g total mass. Inside an Ar filled glovebox (<0.5 ppm of O_2_), Lu pieces (Alfa-Aesar, 99.9%) were turned to filings using the fine side of a high-carbon steel file, weighed, and then added to a 65 cm^3^ stainless-steel grinding vial with two 12.7 mm diameter balls (SPEX). YbH_2_ powder (originally purchased as a Yb powder,^11−13^ American Elements 99.9999%), ZnSb powder (prepared above), and Yb_4_Sb_3_ powder (prepared above) were added to the vial. It was closed, sealed in Mylar, and milled for three rounds of 30 min. Because this reaction consists of powdered reagents most of which are binaries, there is less propensity for cold welding/sticking, and the starting materials already have inherently better elemental distribution. Due to this, the reactions were only scraped between the second and third rounds. Under an inert atmosphere, the reaction was then scraped out of the vial using a chisel and transferred to a 12.7 mm diameter graphite die (CalNano) for reaction and consolidation by spark plasma sintering (SPS). The die was transferred to the chamber of a Dr. Lab Sinter Jr. (Fuji Corp.). SPS instrument, a thermocouple was inserted into the outside of the die, and the chamber was evacuated to <13 Pa and left under active vacuum. The initial pressure was set at 5 kN and the sample was heated to 600 °C, where it was held for 30 min. This step is where the reaction occurs, and the progress can be monitored by the off-gassing of hydrogen from the reaction which causes the chamber pressure to rise.^11^ After dwelling for 30 min, the chamber pressure returns to around the starting value and remains there for the remainder of the reaction/press. For the final consolidation, the reaction was heated to 850 °C for 15 min, and the pressure was increased to 6.5 kN. The resultant black pellets were cleaned of graphite foil using sandpaper and the pellet was sliced with an Isomet Low Speed Cutter diamond saw (Buehler) with cutting fluid for analysis by PXRD. The PXRD data of the products were fully indexed as pure phase Yb_14–*x*_Lu_*x*_ZnSb_11_ until reaching the solubility limit of Lu.

### PXRD Analysis

PXRD was performed on precursors, products, and oxidized materials using a Bruker d8 Advanced Eco on zero background off-axis SiO_2_ plates using Cu K_α1_ and K_α2_ radiation from 15 to 80° 2θ with a step size of 0.01638° 2θ. The resultant patterns were indexed and refined using Rietveld refinement tools in the Jana 2006 software package.^14^

### Elemental Analysis

Scanning electron microscopy (SEM) and energy-dispersive spectroscopy (EDS) were performed on samples set in epoxy using a Thermo Fisher Quattro environmental SEM instrument equipped with a Bruker x-flash 6–100 detector. EDS data and elemental maps were constructed and analyzed by using the Bruker ESpirit software package. A single crystal of Yb_14_MnSb_11_ and a piece of high purity Zn were used as elemental standards for Yb, Sb, and Zn.

### Thermal Conductivity

Thermal diffusivity (*D*) was measured on thin (1–2 mm thick) slices of Yb_14–*x*_Lu_*x*_ZnSb_11_ pellets using a Netzsch LFA 457 Microflash under a flow of high purity Ar with a piece of polished Zr ribbon wrapped around one of the empty position caps to act as an oxygen getter. The thermal conductivity (κ) was determined from the equation: κ = *D* × ρ × *C*_*p*_. The density (ρ) of the pellets was measured by the Archimedes method using toluene as the liquid. All samples had >98% of their theoretical crystallographic densities. The heat capacity (*C*_*p*_) was estimated using the heat capacity of Yb_14_MnSb_11_, which was then converted using the molecular weight of the Yb_14–*x*_Lu_*x*_ZnSb_11_ compound^15^
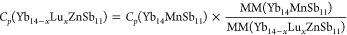


Experimental values for Yb_14_MnSb_11_ were used as an estimation of the coefficient of thermal expansion.^16^ The error in thermal conductivity is estimated to be ±8%, considering the uncertainties from *D*, ρ, and *C*_*p*_.^5^

### Electrical Resistivity

The electrical resistivity of Yb_14–*x*_Lu_*x*_ZnSb_11_ was measured on a custom-built Hall system at the Jet Propulsion Laboratory (JPL). This system measures temperature-dependent electrical resistivity using a Van der Pauw orientation and measuring Hall carrier concentrations and mobilities under a 0.8 T magnetic field.^17^ Errors in electrical resistivity and Hall measurements are estimated to be ±5%.^17^

### Seebeck Coefficient

The Seebeck coefficient of Yb_14–*x*_Lu_*x*_ZnSb_11_ was measured on a custom-built system at JPL. This system utilizes a 2-probe orientation and the light-pipe method to measure the temperature-dependent Seebeck coefficient without exaggeration due to the coldfinger effect.^18,19^ Errors in the Seebeck coefficient using this method can be estimated at ±2%.^18^

### Oxidation Studies

Oxidation studies of Yb_14–*x*_Lu_*x*_ZnSb_11_ (*x* = 0.3) were performed on pieces of dense pellets using thermogravimetry and differential scanning calorimetry (TG/DSC, Netzsch STA 449 Jupiter) under a 50 mL/min flow of dry air (20% oxygen in N_2_, Praxair) in Al_2_O_3_ crucibles from room temperature to 1000 °C. To investigate the oxidation reaction on a larger scale, a pellet of *x* = 0.3 was prepared and after confirmation of purity by PXRD, the pellet was polished and put in a box furnace in an Al_2_O_3_ holder. It was heated at 200 °C/h to 1000 °C and held at that temperature for 12 h in an ambient atmosphere. Half of this oxidized pellet was set in epoxy for SEM imaging of the cross-section. The other half was taken apart, the individual components were ground, and the PXRD was collected and analyzed.

## Results and Discussion

**Figure 2:**
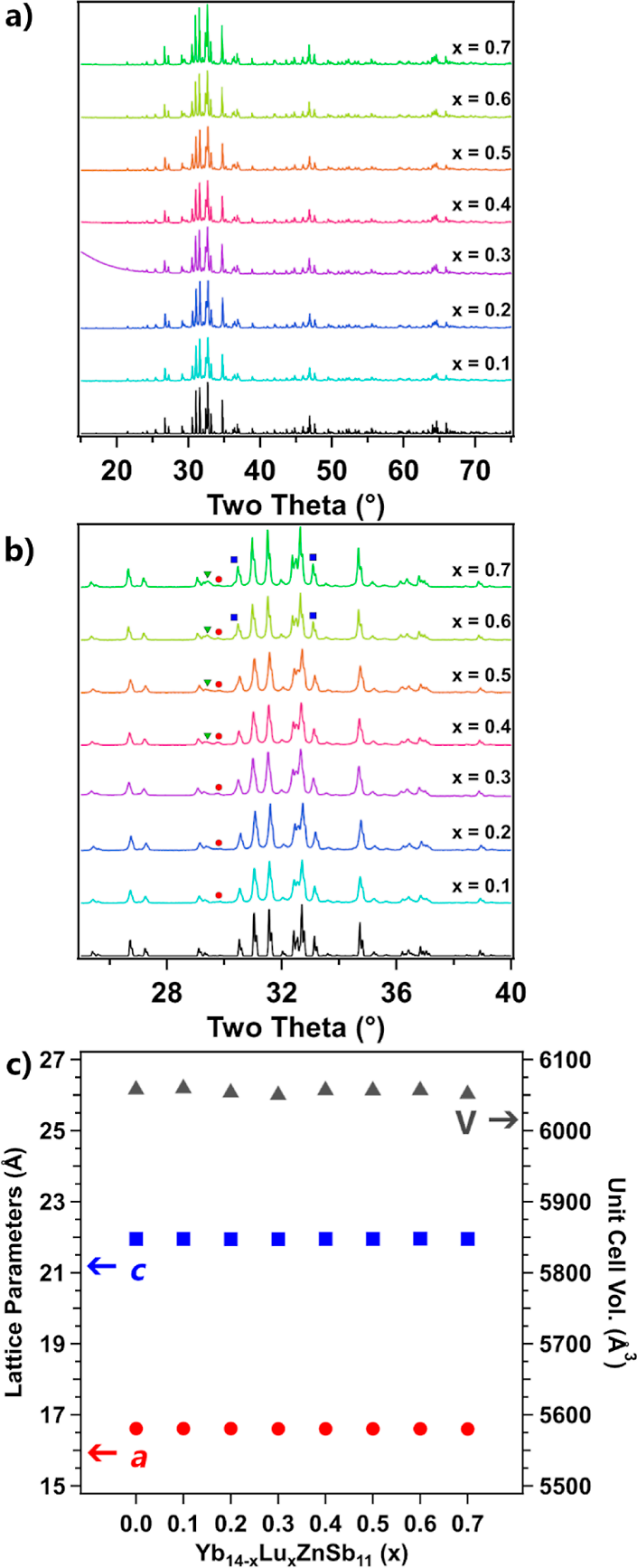
(a) PXRD patterns of the Yb_14–*x*_Lu_*x*_ZnSb_11_ (*x* = 0.1–0.7) series and (b) zoomed-in view of the 25–45° 2θ region. A reference pattern for Yb_14_ZnSb_11_ (ICSD 153158) is shown in black on the bottom, and markers show the reflections for LuSb (green triangles), Yb_2_O_3_ (red circles), and Yb_4_Sb_3_ (blue triangles). (c) Lattice parameters of the Yb_14–*x*_Lu_*x*_ZnSb_11_ series. The *a* (red circles) and *c* (blue squares) parameters are plotted on the left axis, where the unit cell volumes (gray triangles) are plotted on the right.

The full PXRD patterns of the Yb_14–*x*_Lu_*x*_ZnSb_11_ (*x* = 0.1–0.7) series are shown in Figure 2a along with an expanded range in view in Figure 2b. Table 1 shows the results from Rietveld refinement of these PXRD patterns, and the refinements are provided in the Supporting Information, as shown in Figures S1–S7. The samples of *x* = 0.1–0.3 show phase pure Yb_14–*x*_Lu_*x*_ZnSb_11_ aside from a very weak reflection at 29.8° 2θ assigned to Yb_2_O_3_ (<0.9 wt %). At *x* = 0.4, a new reflection assigned to LuSb at 29.4° 2θ appears. Intensities attributed to Yb_4_Sb_3_ at 30.4 and 36.4° 2θ become visible as *x* increases. The reflections of LuSb and Yb_4_Sb_3_ become noticeable as *x* increases due to the solubility limit of Lu within this phase (eq 5). The compositions where the solubility limit is reached agree well with what has been reported for single crystals of Yb_14–*x*_RE_*x*_MnSb_11_ (RE = La–Nd and Sm–Lu), where the solubility limit of most RE elements was found to be *x* ∼ 0.4–0.6.^9,20,21^Equation 5 shows the balanced reaction for a sample of *x* = 0.7, where the solubility of the Lu limit is set at *x* = 0.5.
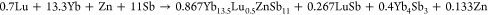
5

**Table 1:** Results from Rietveld Refinement of the Yb_14–*x*_Lu_*x*_ZnSb_11_ PXRD

*x*	Yb_14–*x*_Lu_*x*_ZnSb_11_ wt %	Yb_2_O_3_ wt %	LuSb wt %	Yb_4_Sb_3_ wt %	lattice param. *a*, *c* (Å), *V* (Å^3^)	GoF, *R*_p_, w*R*_p_
0.1	99.50(9)	0.50(13)	0	0	*a* = 16.6132(3)	1.61, 7.25, 9.79
					*c* = 21.9541(5)	
					*V* = 6059.3(2)	
0.2	99.75(5)	0.25(5)	0	0	*a* = 16.6145(3)	1.64, 7.64, 10.23
					*c* = 21.9490(3)	
					*V* = 6053.28(8)	
0.3	99.30(5)	0.70(6)	0	0	*a* = 16.6040(2)	4.23, 2.79, 3.96
					*c* = 21.9450(6)	
					*V* = 6050.1(2)	
0.4	97.50(6)	1.56(6)	0.94(3)	0	*a* = 16.6094(2)	1.56, 5.70, 7.78
					*c* = 21.9554(3)	
					*V* = 6056.8(1)	
0.5	98.20(6)	0.90(5)	0.90(3)	0	*a* = 16.609(2)	1.53, 7.37, 10.31
					*c* = 21.954(2)	
					*V* = 6056.4(1)	
0.6	95.36(9)	0.73(6)	2.29(5)	1.61(12)	*a* = 16.609(1)	1.66, 6.59, 9.12
					*c* = 21.957(1)	
					*V* = 6056.6(7)	
0.7	91.7(2)	0.47(2)	2.75(5)	0.88(4)	*a* = 16.6032(1)	1.53, 6.19, 8.06
					*c* = 21.9522(2)	
					*V* = 6051.5(1)	

Equation 5: the balanced reaction to make Yb_14–*x*_Lu_*x*_ZnSb_11_*x* = 0.7, where the solubility limit of Lu within the main phase is *x* = 0.5. This leads to the formation of LuSb and Yb_4_Sb_3_ to balance the reaction. This reaction is shown using the elements instead of binary precursors for simplicity.

As the solubility limit of Lu is reached, there is free Lu within the reaction mixture that reacts with Sb forming cubic LuSb that is very stable. Because the LuSb phase is more Sb rich than Yb_14–*x*_Lu_*x*_ZnSb_11_, the rest of the reaction is slightly Yb rich, which, in turn, leads to the formation of small amounts of Yb_4_Sb_3_. The lattice parameters of the series are shown in Table 1 and are plotted in comparison with an unsubstituted sample of Yb_14_ZnSb_11_ in Figure 2c. As expected by their similarities in radii, there are no significant changes to the lattice parameters across the series.

**Figure 3:**
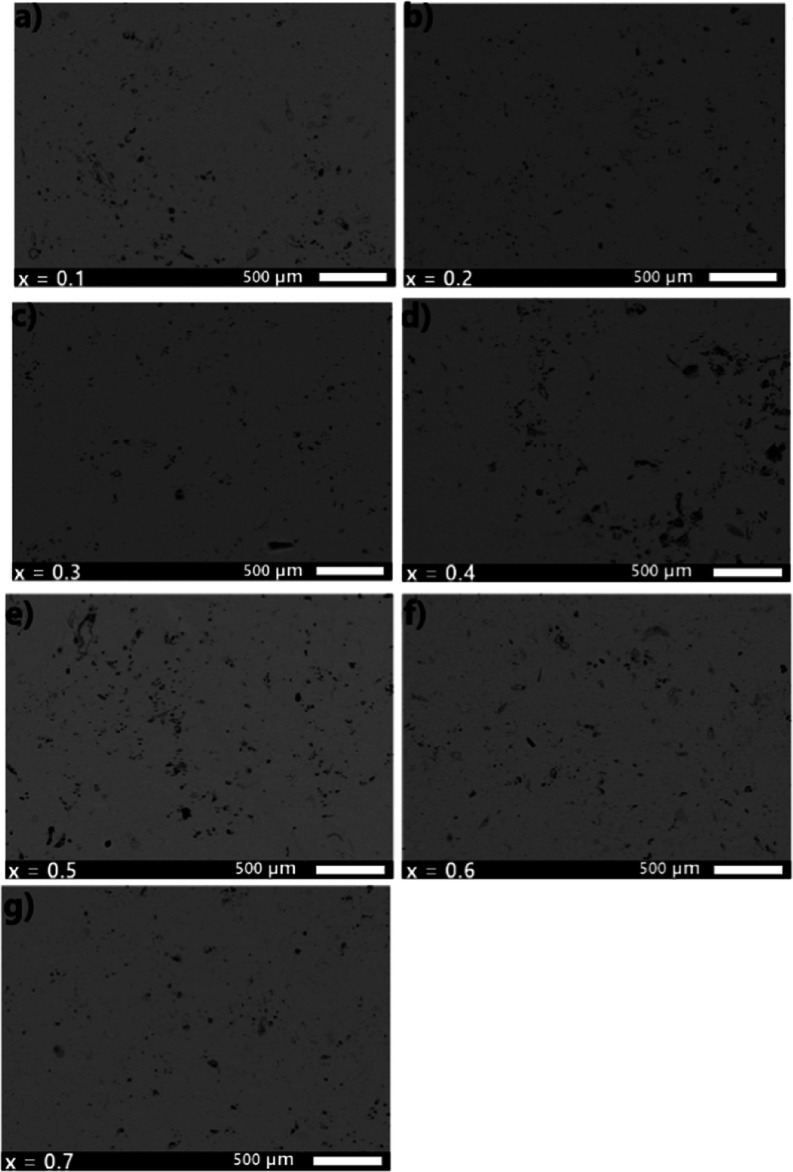
Backscattered electron micrographs of the samples of Yb_14–*x*_Lu_*x*_ZnSb_11_ beginning with *x* = 0.1 in (a) and ending with *x* = 0.7 in (g). The composition, *x*, is indicated at the bottom left with a 500 μm scale bar on the right.

Figure 3 shows the backscattered electron micrographs of Yb_14–*x*_Lu_*x*_ZnSb_11_. Topologically, all samples showed some degree of pullout due to the polishing process, which appears as the black voids on the material’s surface. Samples with lower Lu content seem to show lesser amounts of pullout with *x* = 0.4 and above samples showing much more. Comparing the homogeneity of the contrast across the samples, small dark inclusions can be seen scattered among the main phase. These spots become more numerous and increase in size as *x* increases. This suggests that there is limited incorporation of the Lu into the main Yb_14–*x*_Lu_*x*_ZnSb_11_ phase leading to the formation of the secondary LuSb phase seen in PXRD. The backscattered image and elemental mapping from EDS of Yb_14–*x*_Lu_*x*_ZnSb_11_, *x* = 0.6 along with *x* = 0.2 are provided in Figures S8–S11. Regions of Lu excess correspond to Yb-deficient regions. This supports the hypothesis that the secondary phase forming is likely LuSb rather than Yb_1–*x*_Lu_*x*_Sb as most regions consist of only Lu and Sb.

Table 2 shows the results from EDS on samples of Yb_14–*x*_Lu_*x*_ZnSb_11_ compared to their nominal compositions. All samples are slightly Sb rich and Zn deficient in composition, which can be attributed to the low atomic percent of Zn paired with the overlap in the emission spectrum from Yb, Sb, and Zn, making quantitative deconvolution difficult.^22^ The same can be said about the determination of Lu content, whose spectrum overlaps in energy with Yb, so there is likely to be an increase in the uncertainty within those values. Despite this experimental uncertainty, the lowest Lu content samples (*x* = 0.1, and 0.2) show nominal compositions quite close to that measured by EDS. As *x* increases, the measured Lu content increases as well; however, the amount measured is increasingly less than nominal. Additionally, the standard deviation in the Lu content across the 10 measured points increases significantly as *x* increases. These results further show that Lu is not incorporated beyond *x* ≤ 0.3 and is not homogeneously distributed, instead existing as LuSb inclusions.

**Table 2:** EDS on Pellets of Yb_14–*x*_Lu_*x*_ZnSb_11_ Compared with Nominal Compositions

*x*	Yb %	Yb nom. %	Lu %	Lu nom. %	Zn %	Zn nom. %	Sb %	Sb nom. %
0.1	54.03(34)	53.46	0.43(16)	0.38	1.56(11)	3.85	43.98(32)	42.31
0.2	53.67(43)	53.08	0.54(19)	0.77	1.32(21)	3.85	44.48(26)	42.31
0.3	54.01(44)	52.69	0.59(20)	1.15	1.38(24)	3.85	44.03(43)	42.31
0.4	53.13(57)	52.31	0.55(24)	1.53	1.30(25)	3.85	45.02(60)	42.31
0.5	52.56(2)	51.92	1.16(80)	1.92	1.59(1)	3.85	44.98(1)	42.31
0.6	53.40(62)	51.54	0.66(23)	2.31	1.50(11)	3.85	44.45(52)	42.31
0.7	53.30(1)	51.16	1.05(1)	2.69	1.25(45)	3.85	44.40(68)	42.31

## Thermoelectric Properties

Because the characterization shown above revealed secondary LuSb phases at Lu content *x* > 0.4, the focus of the discussion of the thermoelectric measurements is on Yb_14–*x*_Lu_*x*_ZnSb_11_, *x* = 0.2–0.4. Thermoelectric properties for a sample of *x* = 0.6 compared with *x* = 0, 0.2, 0.3, and 0.4 can be found in Supporting Information, Figure S12.

**Figure 4:**
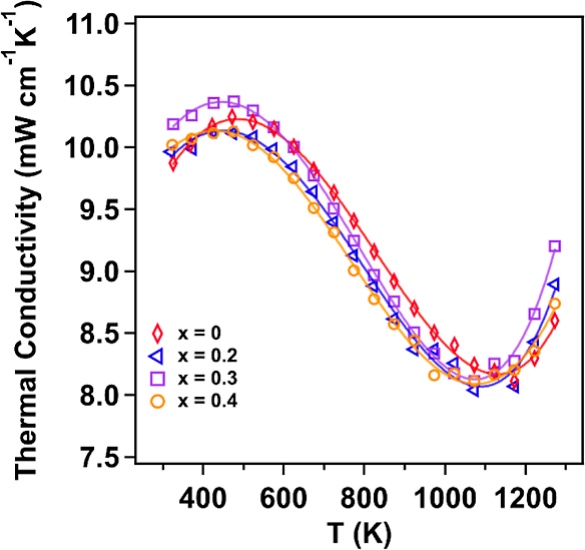
Thermal conductivity of Yb_14–*x*_Lu_*x*_ZnSb_11_ compared to that of an unalloyed sample made by the same route.

Figure 4 shows the thermal conductivity of Yb_14–*x*_Lu_*x*_ZnSb_11_ compared to that of a pristine sample prepared in the same manner. The thermal conductivity follows a typical curve for all compounds of this structure type. At the lowest temperatures, the values of thermal conductivity start at ∼9.9 mW cm^–1^ K^–1^ and the solid solutions are similar in value. The data show a maximum between 423 and 472 K followed by a steady decrease until about 1072–1172 K before increasing again. The distinct “S” shaped thermal conductivity has previously been shown to result from the electronic contribution as carriers move from the Γ band into the degenerate band between N and P, which lies deeper into the valence band and leads to the low-temperature maximum.^15^ After this point, the acoustic scattering of phonons dominates until the onset of bipolar conduction at the highest temperatures, leading to an increased electronic contribution at high temperatures. Looking at where the low-temperature maximum occurs, the *x* = 0.2 and *x* = 0.4 samples show clear shifts to lower temperatures. The maximum of the thermal conductivity for the *x* = 0.3 sample is also likely to be shifted and lie between 423 and 473 K as the thermal conductivities at those two temperatures are within 0.01 mW cm^–1^ K^–1^ of each other. This is outlined in the polynomial model (solid line). As all three alloyed samples show a shift in this low-temperature value, there are two possible reasons for this. The first is a shift of the Fermi level deeper into the valence band (more p-type doping). This is unlikely as Lu is a trivalent cation that replaces either Yb^2+^ or Yb^3+^ in the system and should result in either no change or a lowering of the carrier concentration.^6^ The alternative possibility is that Lu replacing Yb leads to a shift in the relative energies of the light band at gamma and the pocket of degenerate bands between N and P in the electronic band structure. Crystal orbital Hamiltonian population analysis showed that the top valence bands within this structure type are dominated by Yb–Sb bonding and Sb–Sb states with slight antibonding characters.^5^ As Lu is only replacing Yb and does not significantly change the unit cell size, it should lead to a slight shift of the Yb–Sb states while leaving the Sb–Sb states untouched. The high temperature increase is also a characteristic of the electronic band structure and should provide clues about the potential changes. In all three alloyed samples, there is a shift in the onset of this high-temperature event, which can be attributed to the point at which minority carriers (electrons) have enough energy to cross the band gap into the conduction band. For a p-type material, the temperature at which this onset occurs is related to both the band gap and the location of the Fermi level relative to the valence band edge. To effectively determine which of these possibilities could lead to this trend, the electronic transport data presented below will be needed.

**Figure 5:**
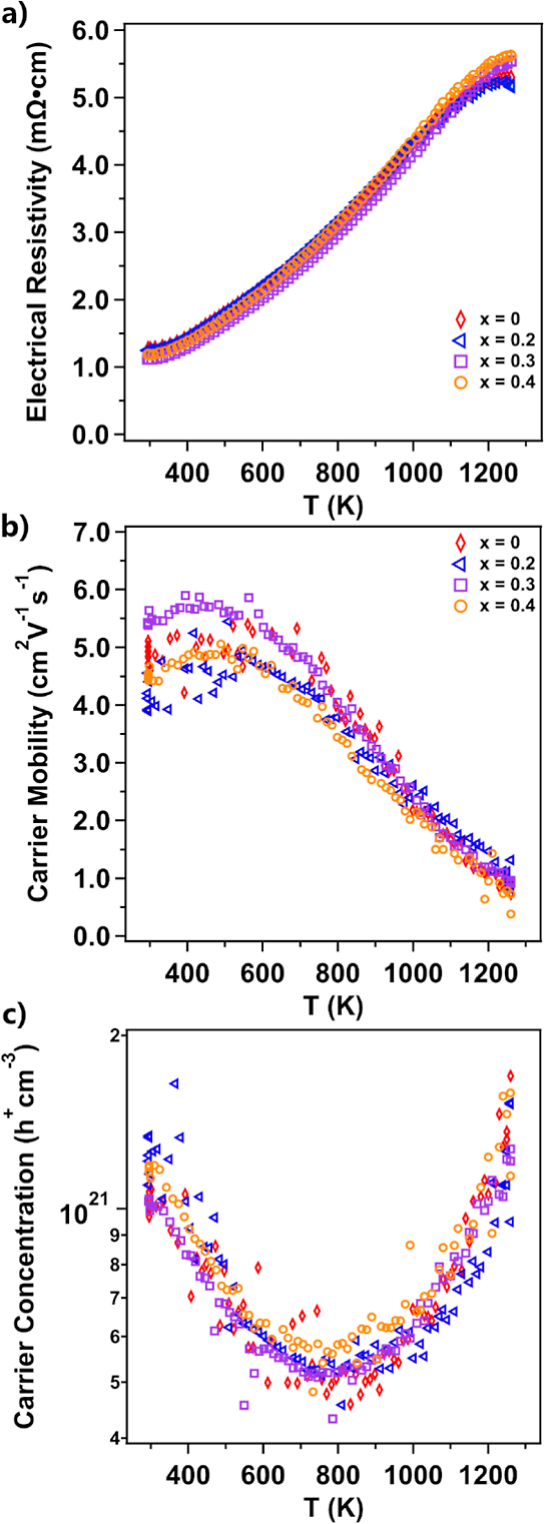
(a) Electrical resistivity, (b) carrier mobility, and (c) Hall carrier concentration of the Yb_14–*x*_Lu_*x*_ZnSb_11_ series.

Figure 5 shows the (a) electrical resistivity, (b) mobility, and (c) Hall carrier concentration carrier of the Yb_14–*x*_Lu_*x*_ZnSb_11_ series. The samples are quite similar apart from *x* = 0.4. The electrical resistivity starts at ∼1.3 mΩ·cm at 295 K and the substituted samples are slightly lower, 1.2–1.1 mΩ·cm. The resistivity steadily increases until reaching a maximum at about 1255 K after which there is a rollover due to bipolar conduction.^19^ The carrier mobility of the pristine sample and the *x* = 0.2 composition show very similar behavior across the entire temperature range beginning at ∼5 cm^2^ V^–1^ s^–1^ from room temperature to ∼500 K after which the carrier mobility steadily decreases to a value around 1.0 cm^2^ V^–1^ s^–1^ at 1260 K. The sample with *x* = 0.3 shows a slight increase in the room-temperature mobility with values around 5.5 cm^2^ V^–1^ s^–1^ from room temperature to ∼500 K. After this point, it also steadily decreases to the same high temperature value as the other samples. The carrier mobility of *x* = 0.4 begins at a lower value of 4.6 cm^2^ V^–1^ s^–1^ and much like the other samples shows a drop in mobility after ∼500 K. The carrier concentrations of the pristine, *x* = 0.2 and *x* = 0.3 samples all show similar values and temperature-dependent behavior, beginning around 1.03–1.24 × 10^21^ h^+^ cm^–3^ and decreasing to ∼5 × 10^20^ h^+^ cm^–3^ at 735–815 K after which there is an increase in the carrier concentration as minority carriers begin to excite over the band gap. Upon introduction of the metallic LuSb impurity in *x* = 0.4, there is little effect on the carrier concentrations and they are comparable to *x* = 0.2 and 0.3. A table of room-temperature carrier concentration and mobility values can be found in the Supporting Information (Table S1).

**Figure 6:**
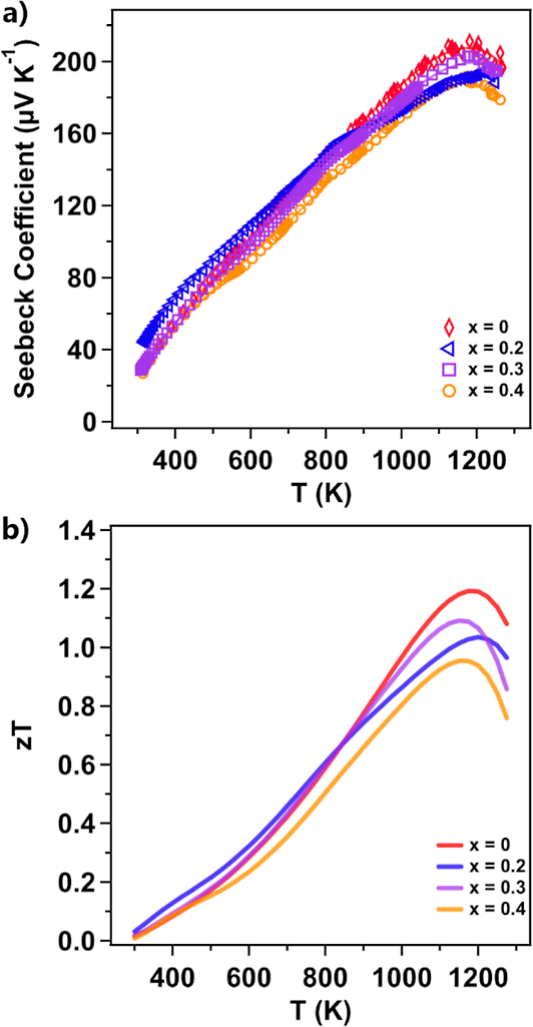
(a) Seebeck coefficient and (b) *zT* of Yb_14–*x*_Lu_*x*_ZnSb_11_.

Figure 6a shows the Seebeck coefficient of the Yb_14–*x*_Lu_*x*_ZnSb_11_ series. The pristine sample begins at a value of 32.27 μV K^–1^ at 311.64 K and then increases with the temperature to a maximum of 210.13 μV K^–1^ at 1203.66 K after which the Seebeck coefficient decreases due to the contribution of minority carriers. The Seebeck coefficients of the Lu-substituted samples all begin at similarly low values at room temperature and follow the same general temperature dependence as pure Yb_14_ZnSb_11_. However, at the highest temperatures, the samples of Yb_14–*x*_Lu_*x*_ZnSb_11_ show reductions in the maximum Seebeck coefficient, reaching 194.29 μV K^–1^ at 1223.4 K, 203.16 μV K^–1^ at 1175.97 K, and 190.12 μV K^–1^ at 1179.86 K for *x* = 0.2, 0.3, and 0.4, respectively. The Seebeck for the *x* = 0.2 is slightly out of line with the other compositions above 800 K and this might be attributed to crystallographic defects or the presence of an unidentified impurity. Using the value and temperature of the maximum Seebeck coefficient, the Goldsmid–Sharp method of band gap determination can be applied.^23^ From this analysis, the pristine sample has a band gap of 0.505 eV, *x* = 0.2 has a band gap of 0.475 eV, *x* = 0.3 has a band gap of 0.478 eV, and *x* = 0.4 has a band gap of 0.448 eV. This suggests that Lu-substitution reduces the band gap of the material. Lu^3+^ is smaller than Yb^2+^ and more electronegative, which may explain the reduction in the band gap.

The unitless thermoelectric figure of merit for the samples of Yb_14–*x*_Lu_*x*_ZnSb_11_ are provided in Figure 6b. The sample of Yb_14_ZnSb_11_ reaches a peak *zT* of 1.19 between 1175–1200 K. As Lu is added, the low-temperature *zT* remains unchanged, but at high temperatures the samples of *x* = 0.2, *x* = 0.3, and *x* = 0.4 show reductions in peak *zT* reaching 1.04 at 1200 K, 1.09 at 1150 K, and 0.955 at 1150 K, respectively. This affect is attributed to the lower Seebeck coefficients at higher temperatures observed in these samples, and the *zT* reflects the Seebeck trend.

## Oxidation Studies

To better understand the role of Lu alloying on the oxidation properties of Yb_14–*x*_Lu_*x*_ZnSb_11_*x* = 0.3 was chosen. This was the most Lu rich composition that did not show the presence of LuSb.

**Figure 7:**
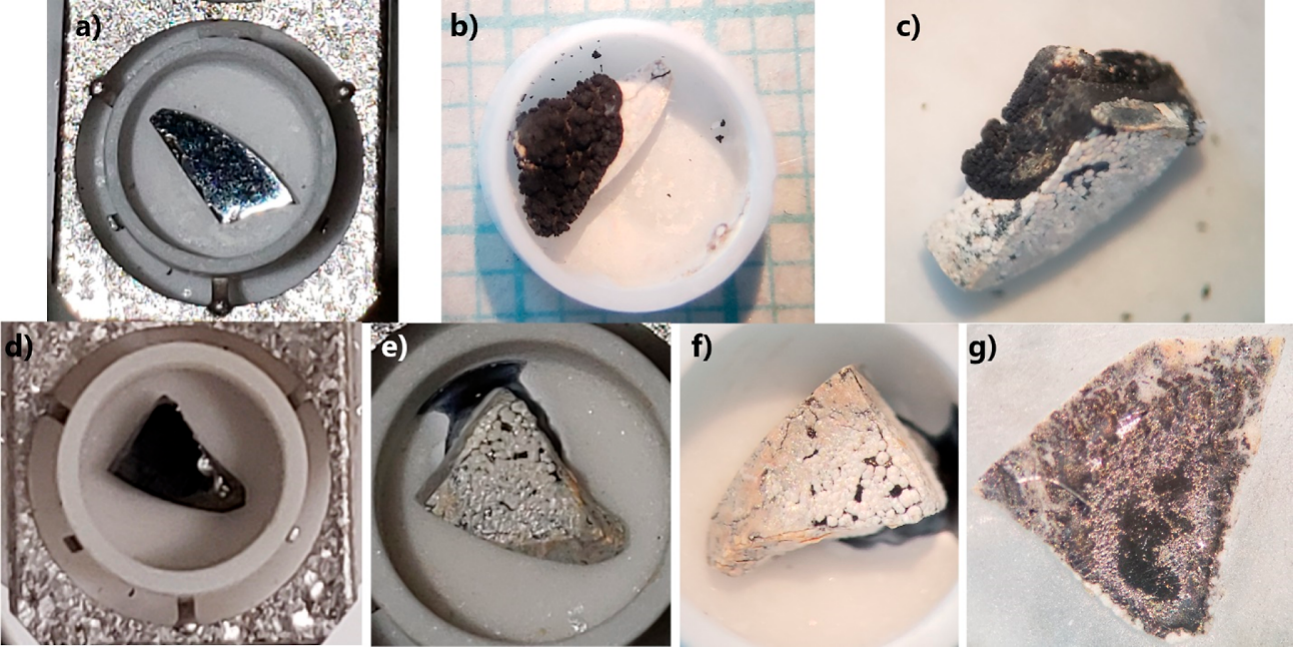
Images of two pieces of a Yb_14–*x*_Lu_*x*_ZnSb_11_ (*x* = 0.3) pellet (a,d) before and (b,c,e,f) after oxidation along with (g) cross-section of the bottom sample.

Figure 7 shows images of two pieces of the same Yb_14–*x*_Lu_*x*_ZnSb_11_ (*x* = 0.3) pellet before and after oxidation. Both samples started as polished pieces of a black metallic material. After oxidation, the sample shown in Figure 7a developed a bright white outer layer with a black growth of bulbous black material (Figure 7b,c). The underside of the sample shows the white shell growing in small circular patches with a black material appearing in between. The second sample showed a much less continuous outer layer, instead showing the same circular patterning seen at the bottom of the first sample. In this second case, the black material extruding from the sample appears as a black film that has reacted with the Al_2_O_3_ crucible, fusing the sample. The black film is likely YbSb_2_, similar to what was seen in the oxidation of unalloyed Yb_14_ZnSb_11_.^8^ The difference in the appearance of this material may be due to reactions with Al_2_O_3_ seen in the second piece. After breaking the piece free from the crucible, the second sample was set in epoxy and sanded to reveal the cross-section, as shown in Figure 7g. The cross-section shows regions of a solid white outer layer with small islands of a black metallic material. Streaks of white continue through the metallic material toward the interior of the sample but stop before the core region. The sample’s core consists of a porous region surrounding a solid center.

**Figure 8:**
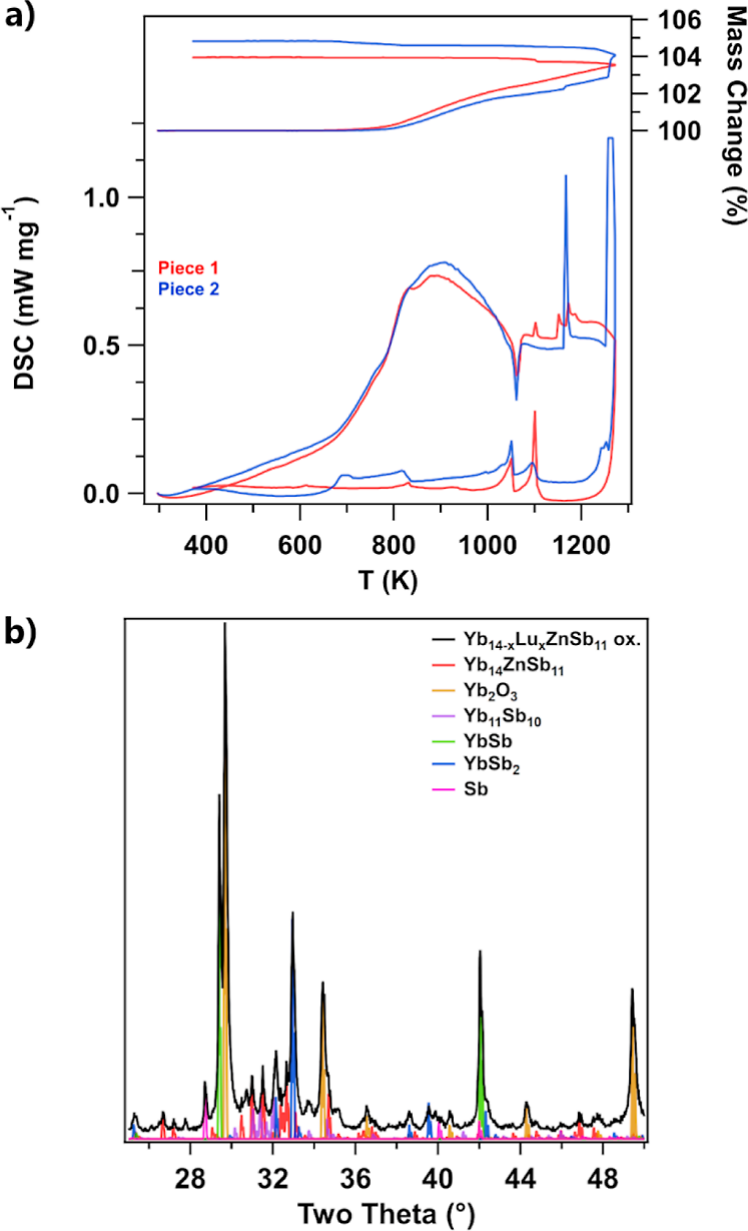
(a) TG (top, right axis) and DSC (bottom, left axis) of pieces of Yb_14–*x*_Lu_*x*_ZnSb_11_ from room temperature to 1173 K under dry air and (b) PXRD pattern of one of the oxidized samples. The experimental diffraction pattern is shown in black with reference colors for each phase in their respective colors.

Figure 8a shows the TG/DSC from the oxidation of both pieces of Yb_14–*x*_Lu_*x*_ZnSb_11_ from room temperature to 1273 K and Figure 8b provides the PXRD pattern of the top (Figure 7a–c) sample after oxidation. In the TG trace, both pieces show no significant mass gain (>0.1%) until 785 K, after which a rapid mass gain begins to lessen in slope as temperature increases. The mass of the first piece (see Figure 7a) smoothly increases to 103.55% at 1273 K, whereas the second piece (Figure 7d) has two rapid gains in mass corresponding to events seen in the DSC. On cooling, the first piece has a single small gain in mass, corresponding to an event in the DSC. Even with this small gain, it only increased 0.40% in mass on cooling, reaching a final 103.95% of its original mass. The second piece shows a gradual increases in mass upon cooling corresponding to a sweeping event in the DSC. This piece gained 4.36% in mass on heating and reached a final mass of 104.83% of its starting mass after cooling. In the DSC of both samples, a sweeping exotherm is observed on heating which increases in slope at 700 K. This exotherm reaches a peak at 900 K, gradually decreasing in magnitude as temperatures increase. This sweeping exotherm is attributed to oxidation that increases in rate with temperature and diminishes as a more passivating oxide layer forms. As temperatures increase to 1061 K, there is a sharp endotherm exhibited by both pieces with a corresponding exotherm in cooling. This event is likely the melting and solidification of the black material extruded from both pieces. The first piece shows a small exotherm at 1100 K on heating and cooling. At the highest temperatures, the first piece shows a group of three exotherms between 1150 and 1175 K. The second piece shows two very strong exotherms at 1175 and 1270 K, the higher one of which has been trimmed to better accommodate the lower temperature signals (a full version of the plot can be found in Supporting Information, Figure S13). These events at the highest temperatures likely correspond to reactions between the black molten material and the Al_2_O_3_ crucible or the oxidative environment. The second piece shows an additional broad exotherm between 675 and 830 K, which may correspond to additional reactions related to the extruded material.

Figure 10b shows the PXRD pattern of the top (Figure 7a–c) sample after oxidation. The diffraction pattern shows intense reflections from both YbSb and Yb_2_O_3_. The YbSb diffraction pattern is shifted slightly, likely due to some incorporation of Lu into the structure. The other intense reflection is due to the Sb-rich phase YbSb_2_. Less intense reflections can be assigned to Yb_11_Sb_10_, Sb, and Yb_14–*x*_Lu_*x*_ZnSb_11_. This result is similar to what was seen in the oxidation of Yb_14_ZnSb_11_, where the Yb and Zn-formed oxides, leading to the formation of more Sb rich Yb–Sb binary phases. However, there is the notable inclusion of the phase YbSb, which was not seen in the oxidation of the pristine composition. The YbSb phase is likely stabilized by the trivalent Lu cation, which helps the compound to be closer to charge balanced.

**Figure 9:**
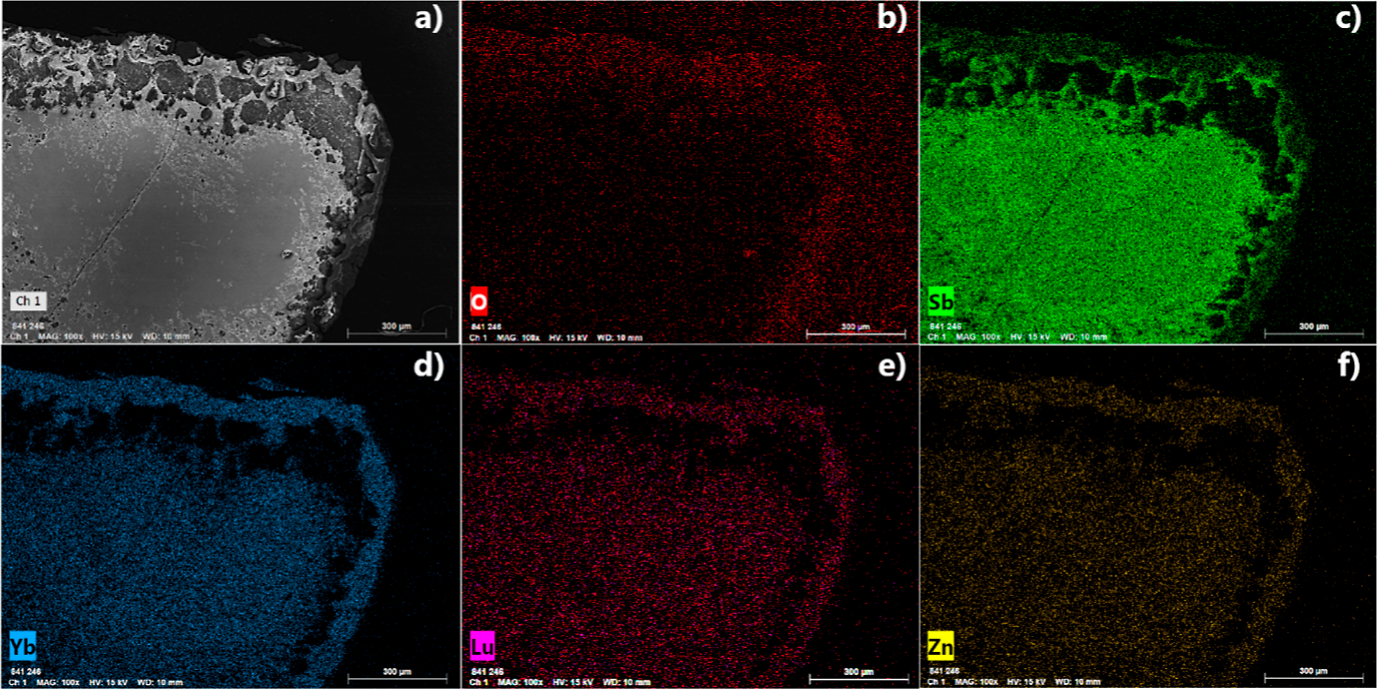
(a) SEM and elemental maps of (b) O (red), (c) Sb (green), (d) Yb (blue), (e) Lu (pink), and (f) Zn (gold) for the second piece of Yb_14–*x*_Lu_*x*_ZnSb_11_ (*x* = 0.3) after oxidation. The elements are indicated on the bottom left, and the scale bars, indicated on the bottom right, are all 300 μm.

**Figure 10:**
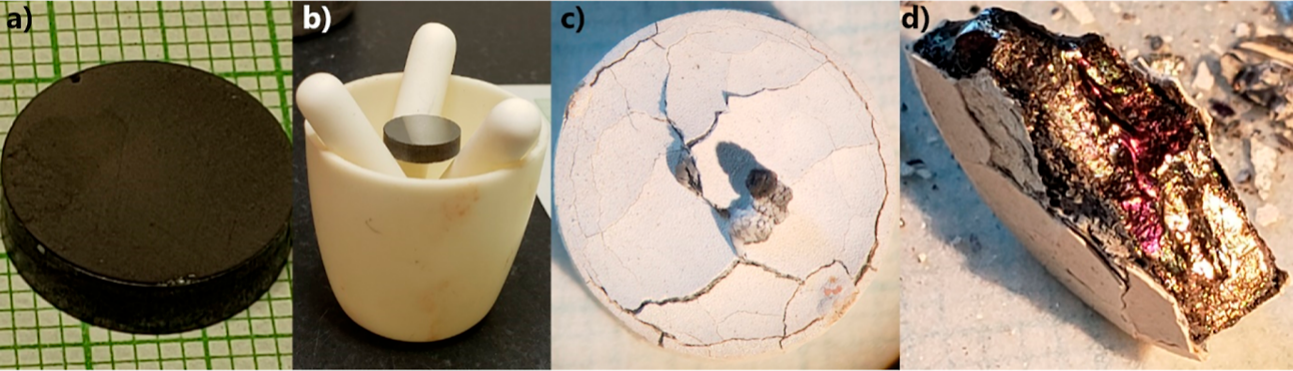
Pictures of the Yb_14–*x*_Lu_*x*_ZnSb_11_ (*x* = 0.3) pellet (a,b) before and (c) after. (d) Cross-section of the pellet after oxidation.

Figure 9 shows the elemental mapping from EDS on the second piece of Yb_14–*x*_Lu_*x*_ZnSb_11_ (*x* = 0.3) after oxidation. The SEM micrograph and elemental maps reveal that the sample consists of an outer layer that is oxygen rich with relatively low concentrations of Sb. Inside is a porous region, with streaks of mostly Sb and Yb running through. The interior shows an even distribution of Yb, Lu, Zn, and Sb with relatively low concentrations of O concentrations. EDS on points of the outer layer was consistent with Yb_2_O_3_ with small amounts of Sb, Zn, and Lu. Points within the porous region have elemental compositions consistent with YbSb_2_ and with increasing points of YbSb moving closer to the interior. The core of the sample shows only 3 at. % of O with Yb, Lu, Sb, and Zn contents close to that of Yb_14–*x*_Lu_*x*_ZnSb_11_.

To investigate how oxidation occurs on a larger scale, an entire pellet (12.7 mm diameter, ∼3 mm thick) was polished and placed within a Al_2_O_3_ holder, which minimizes the contact between the Al_2_O_3_ and the sample. Figure 10 shows the pellet of Yb_14–*x*_Lu_*x*_ZnSb_11_ (*x* = 0.3) before (a, b) and after (c, d) being at 1000 °C for 12 h under ambient atmospheric conditions. The pellet begins with a highly polished black metallic surface. After oxidation, the sample has developed a glossy bright white outer layer, which shows some cracking. Along with that, there is the same black bulbous growth of material coming from the cracks on the surface that was seen in one sample during TG/DSC. This material appears more white/gray than what was seen in the TG/DSC likely due to more oxidation. When the sample was broken in half (Figure 9d), the outer layer remained adhered to the inner core of the sample. The interior consisted of porous region directly below the white outside. The very core was a solid pellet of black material, which showed an iridescent coloring depending on the direction of the lighting. This suggests that the material in the core may be slightly oxidized. In comparison to the oxidation of Lu-substituted Yb_14_MnSb_11_, the sample here shows significantly less material, which has melted out of the sample and the outer oxide coating shows better adherence to the inner core.^10^

**Figure 11:**
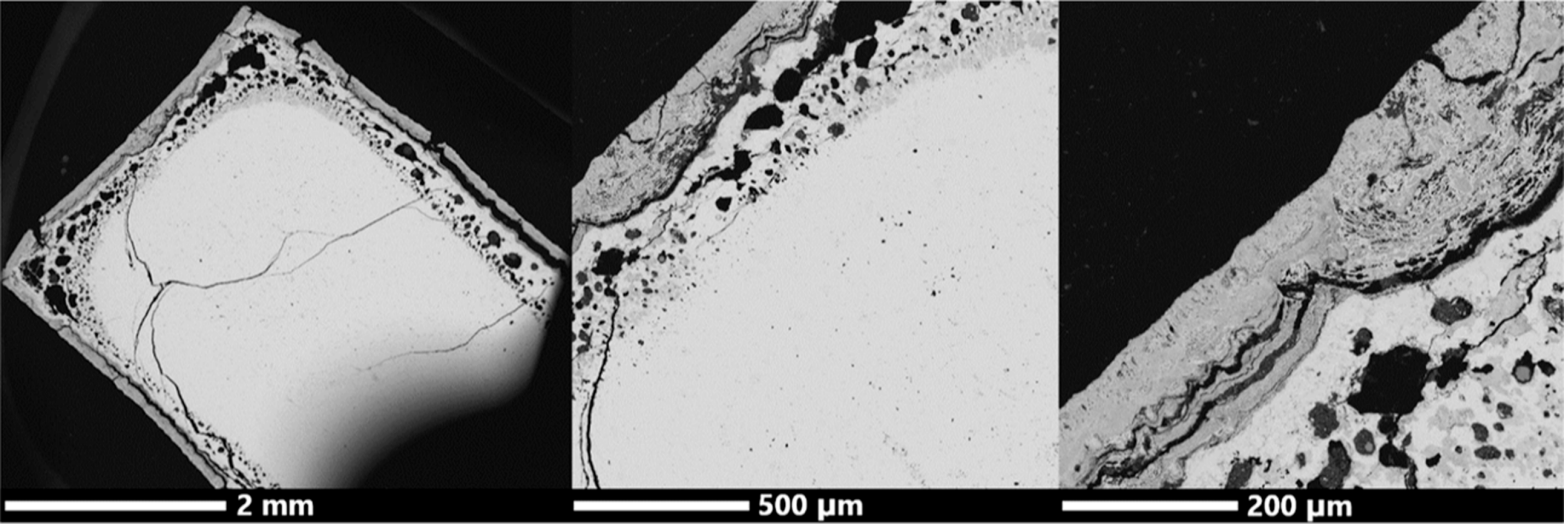
Backscattered electron micrographs of the cross-section of the oxidized Yb_14–*x*_Lu_*x*_ZnSb_11_ pellet with (a) showing an overall view at low magnification, (b) showing the sample at higher magnification, and (c) showing a zoomed-in view of the outer layer.

Figure 11 shows backscattered electron micrographs of the cross section of the oxidized Yb_14–*x*_Lu_*x*_ZnSb_11_ pellet with (a) showing an overall view at low magnification, (b) showing the sample at higher magnification, and (c) showing a zoomed in view of the outer layer. The sample consists of a darker outer region, which shows some cracking, and is an average of 50–100 μm thick in most regions, although it extends to ∼200 μm in others. Inside of this layer is a porous region. which is closer in contrast to the core, although it does show some differences in contrast throughout. The porosity in this region is likely due to the material which melted out of the sample and shows less porosity than oxidized Yb_14–*x*_Lu_*x*_MnSb_11_.^10^ The core shows a consistent lighter contrast, suggesting that it is made of a higher average *Z* material than the outer shell.

**Figure 12:**
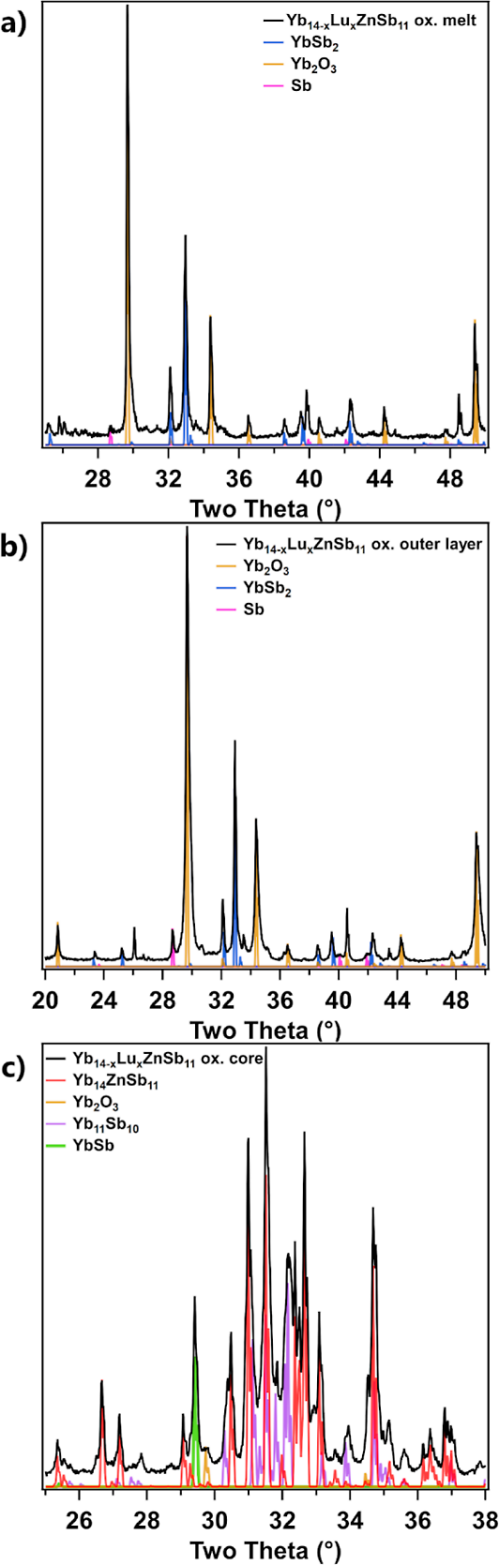
PXRD patterns of the (a) material that melted from the sample, (b) outer layer, and (c) inner core of the oxidized pellet of Yb_14–*x*_Lu_*x*_ZnSb_11_ (*x* = 0.3). Contributions of various compounds are indicated with color.

Figure 12a shows the PXRD pattern from the black, bulbous material extruded from the sample. The PXRD can be indexed as a mixture of YbSb_2_, Yb_2_O_3_, and Sb. The melting of YbSb_2_ after it was produced during oxidation was also seen in the pristine compound. The Yb_2_O_3_ and Sb are likely to result from the oxidation of YbSb_2_ after it extruded through the outer layer of the oxidizing pellet. The lack of any YbZnSb_2_ phase seen in the molten material presents a key advantage of the Yb_14_ZnSb_11_ system over Yb_14_MnSb_11_ which was observed to have a YbMnSb_2_–YbSb_2_ eutectic upon oxidation.^8,10^Figure 12b shows the diffraction pattern from the white outer layer of the oxidized pellet. This layer consists mostly of Yb_2_O_3_ with small amounts of YbSb_2_ and Sb. This suggests that this layer forms from the oxidation of YbSb_2_ which forms Yb_2_O_3_ and Sb. The Sb then acts as a flux before further oxidizing and sublimating as Sb_4_O_6_.^24^ This would help to explain the glossy appearance of the outer layer. The PXRD pattern from a portion of the inner core is shown in Figure 12c. The pattern shows reflections that can be indexed as Yb_14_ZnSb_11_, Yb_11_Sb_10_, YbSb, and a very weak reflection from Yb_2_O_3_.

Because the core consists of starting material, Yb_2_O_3_, and the S-rich binaries Yb_11_Sb_10_, and YbSb, and further out in the sample consists of YbSb_2_, Yb_2_O_3_, and Sb, the oxidation of this material likely occurs in a similar series of reactions as postulated for the pristine compositions Yb_14_MSb_11_ (M = Mn, Mg, and Zn).^8,10^Equations 6–10 show a possible pathway that would explain the above oxidation. Lu is left out for simplicity and should be assumed to be present wherever Yb appears due to the chemical similarity.
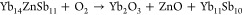
6
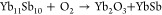
7
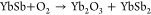
8
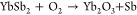
9
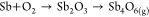
10

Equations 6–10: a possible pathway for the oxidation of Yb_14–*x*_Lu_*x*_ZnSb_11_.

## Conclusions

Lu-substitution in Yb_14–*x*_Lu_*x*_ZnSb_11_ to *x* ∼ 0.3 was achieved using binary precursors in a similar manner as reported for Yb_14_ZnSb_11_.^11^ As excess Lu was added to the system, LuSb, and, in turn, Yb_4_Sb_3_ formed to keep the reaction balanced. As Lu was added, the thermoelectric figure of merit decreased due to a reduction in the Seebeck coefficient, attributed to a smaller band gap. The effect of Lu-substitution in Yb_14_ZnSb_11_ is in contrast to that observed for Yb_14_MnSb_11_ and is attributed to differences in the Yb valency.^6,10^ The introduction of Lu into the system helped stabilize the YbSb phase upon oxidation; however, it did not negate the formation and subsequent melting of YbSb_2_, which proves to be problematic for forming a continuous passivating oxide shell at the highest temperatures. Even with the melting of YbSb_2_, the core of the sample after 12 h at 1000 °C in an ambient atmosphere showed a large amount of Yb_14–*x*_Lu_*x*_ZnSb_11_ remained. It may be possible that under less oxygen-rich atmospheres or reduced temperatures this system may form a passivating oxide shell, which could be utilized for sublimation protection or protection from mildly oxidizing operating conditions.

## Supporting Information Available

Rietveld refinement of Yb_14–*x*_Lu_*x*_ZnSb_11_; backscattered SEM micrographs for Yb_14–*x*_Lu_*x*_ZnSb_11_, *x* = 0.2, 0.6; EDX elemental mapping for Yb_14–*x*_Lu_*x*_ZnSb_11_, *x* = 0.2, 0.6; thermoelectric properties of Yb_14–*x*_Lu_*x*_ZnSb_11_, *x* = 0, 0.2, 0.3, 0.4, 0.6; table of room-temperature Hall mobility and carrier concentration values; and TG/DSC graph for two pieces of Yb_14–*x*_Lu_*x*_ZnSb_11_ (*x* = 0.3) from room temperature to 1173 K under a 50 mL/min flow of dry air (PDF)
