# Supplementary material for: Impact of Lu-Substitution in Yb14–xLuxZnSb11: Thermoelectric Properties and Oxidation Studies
Source: ACS Appl Energy Mater. 2023 Oct 4;6(20):10628–38. doi: 10.1021/acsaem.3c01756 (PMC10598619; doi:10.1021/acsaem.3c01756)
Supplement: Supplementary file 1 — ae3c01756_si_001.pdf [file ae3c01756_si_001.pdf]

Supporting Information

The Impact of Lu Substitution in $Yb_{14-x}Lu_xZnSb_{11}$: Thermoelectric Properties and Oxidation Studies

Andrew P. Justl[1], Logan Winston[1], Sabah K. Bux[2], and Susan M. Kauzlarich[1*]

1: *Department of Chemistry, One Shields Ave, University of California, Davis, CA 95616 USA*

2: *Thermal Energy Conversion Technologies Group, Jet Propulsion Laboratory, California Institute of Technology, 4800 Oak Grove Drive, MS 277-207, Pasadena, CA 91109*

*Corresponding author: smkauzlarich@ucdavis.edu

Contents

Figures S1-7: Rietveld refinement of the $Yb_{14-x}Lu_xZnSb_{11}$, x = 0.1, 0.2, 0.3, 0.4, 0.5, 0.6, 0.7 PXRD patterns

Figure S8: Backscattered electron micrograph of $Yb_{14-x}Lu_xZnSb_{11}$, x = 0.2

Figure S9: Elemental maps of $Yb_{14-x}Lu_xZnSb_{11}$, x = 0.2

Figure S10: Backscattered electron micrograph of $Yb_{14-x}Lu_xZnSb_{11}$, x = 0.6

Figure S11: Elemental maps of $Yb_{14-x}Lu_xZnSb_{11}$, x = 0.6

Figure S12: Thermoelectric properties of $Yb_{14-x}Lu_xZnSb_{11}$, x = 0, 0.2, 0.3, 0.4, 0.6

Table S1. Room Temperature Hall Mobility and Carrier Concentration Values for $Yb_{14-x}Lu_xZnSb_{11}$

Figure S13: The complete TG trace (top, right axis) and DSC (bottom, left axis) for two pieces of $Yb_{14-x}Lu_xZnSb_{11}$ (x = 0.3) from room temperature to 1173 K under a 50 mL/ min flow of dry air

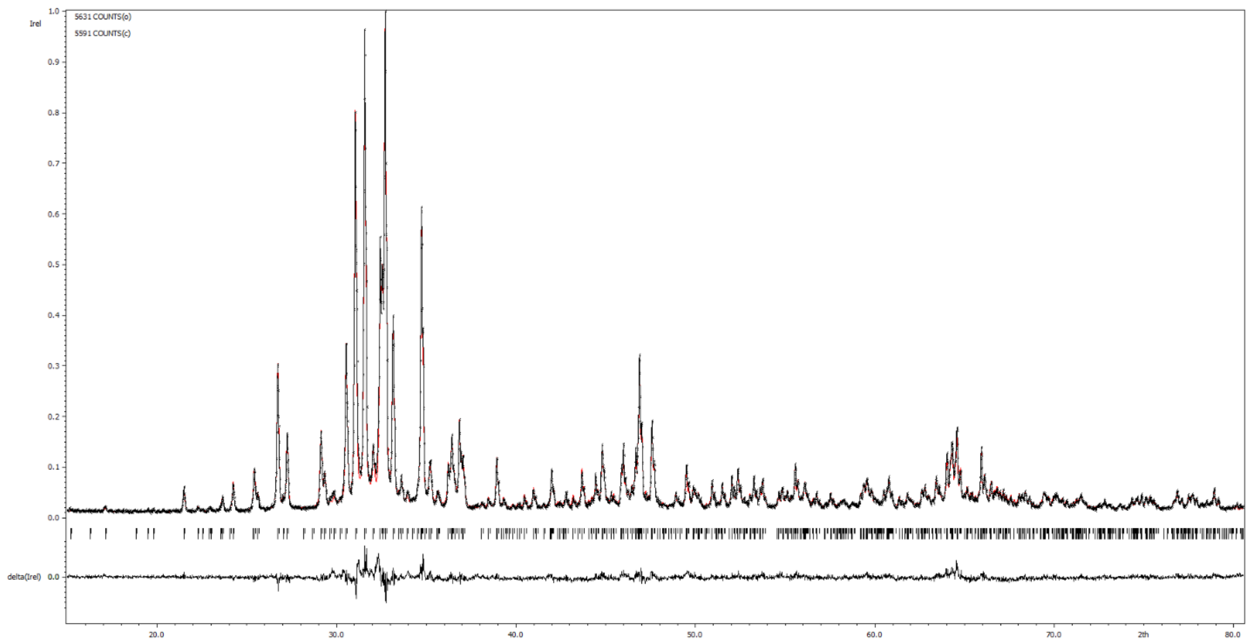

Figure S1: Rietveld refinement of the $Yb_{14\text{-}x}Lu_xZnSb_{11}$ $x = 0.1$ PXRD pattern.

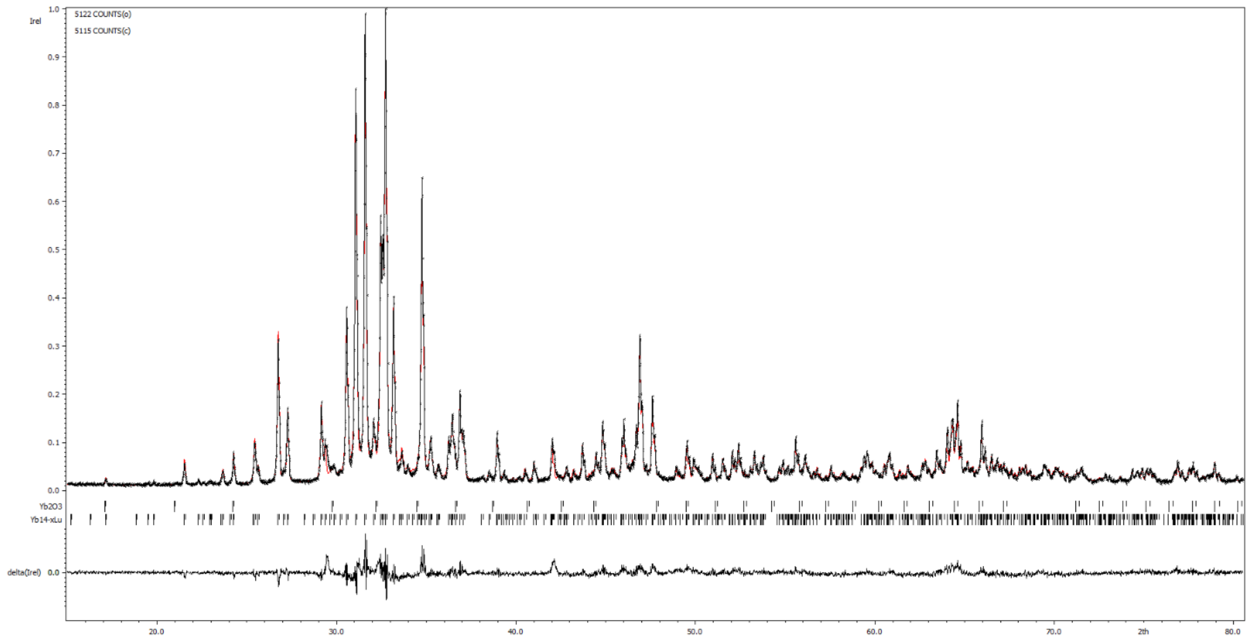

Figure S2: Rietveld refinement of the $Yb_{14\text{-}x}Lu_xZnSb_{11}$ $x = 0.2$ PXRD pattern.

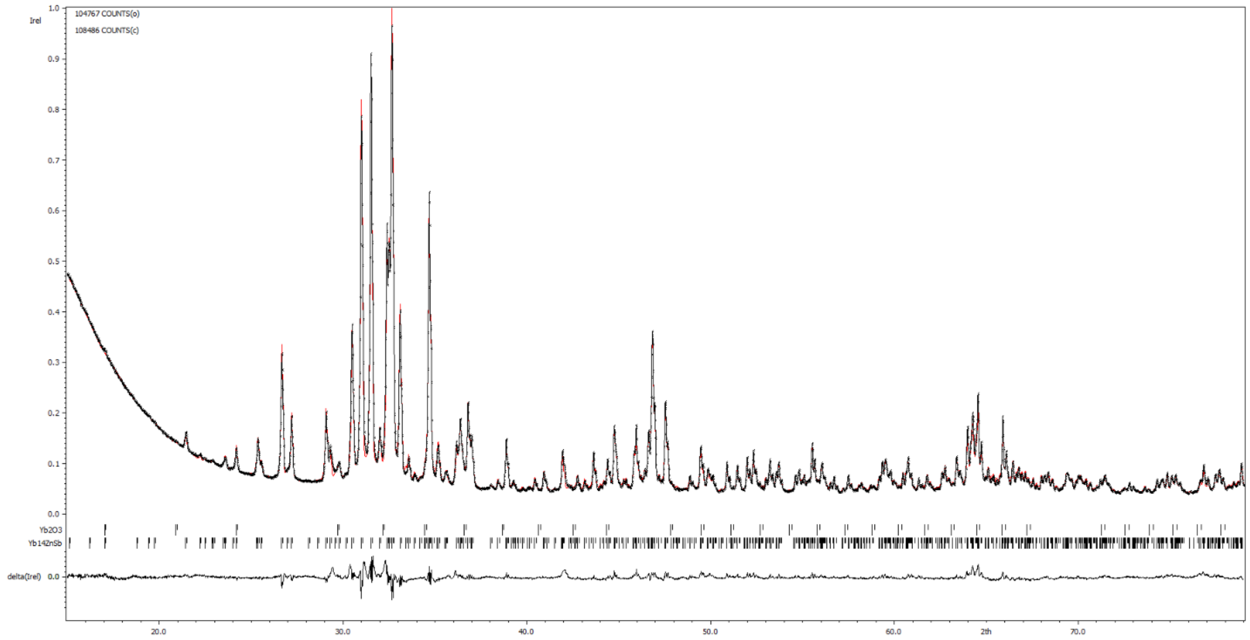

Figure S3: Rietveld refinement of the $Yb_{14-x}Lu_xZnSb_{11}$ $x = 0.3$ PXRD pattern.

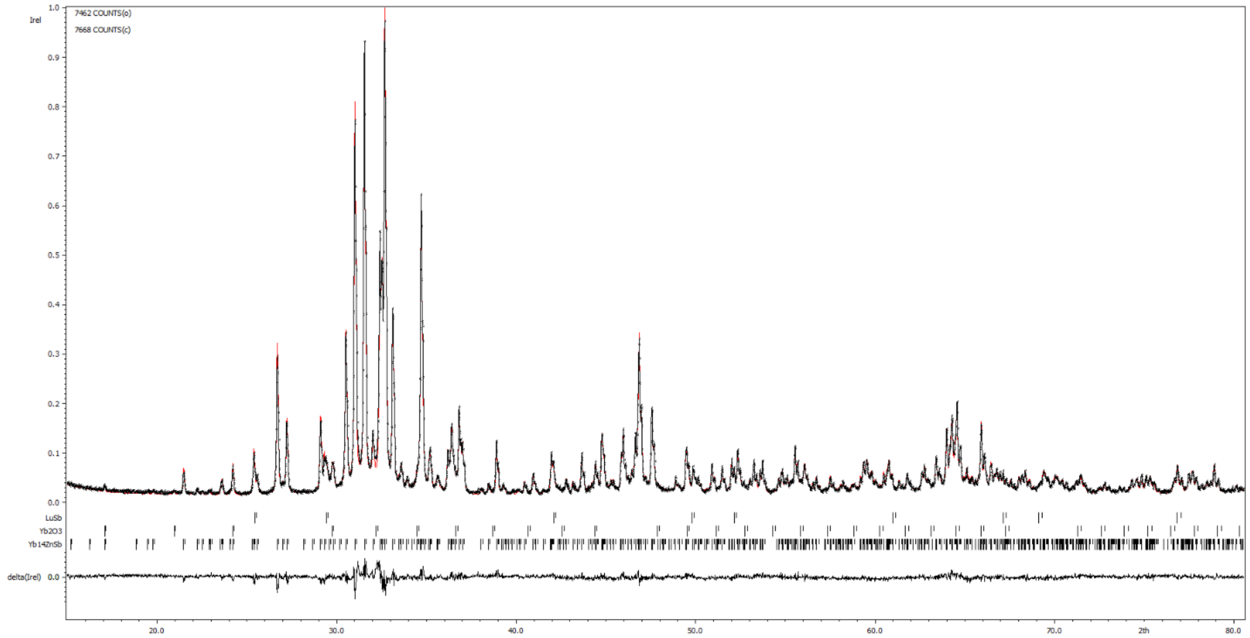

Figure S4: Rietveld refinement of the $Yb_{14-x}Lu_xZnSb_{11}$ $x = 0.4$ PXRD pattern.

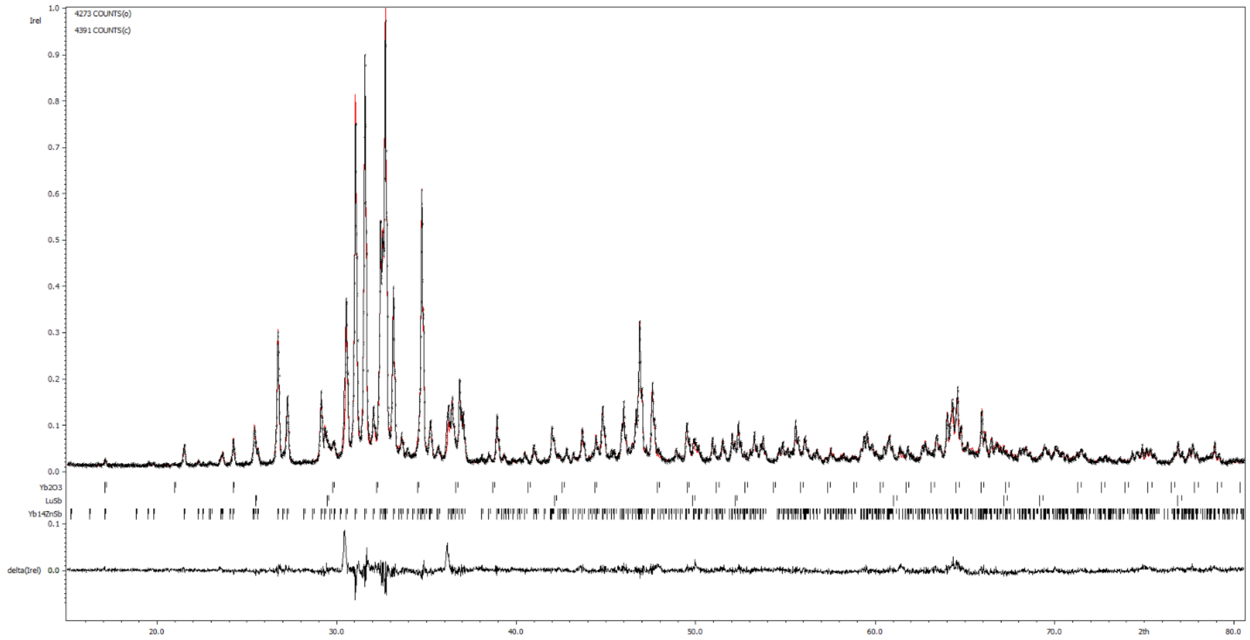

Figure S5: Rietveld refinement of the $Yb_{14-x}Lu_xZnSb_{11}$ $x = 0.5$ PXRD pattern.

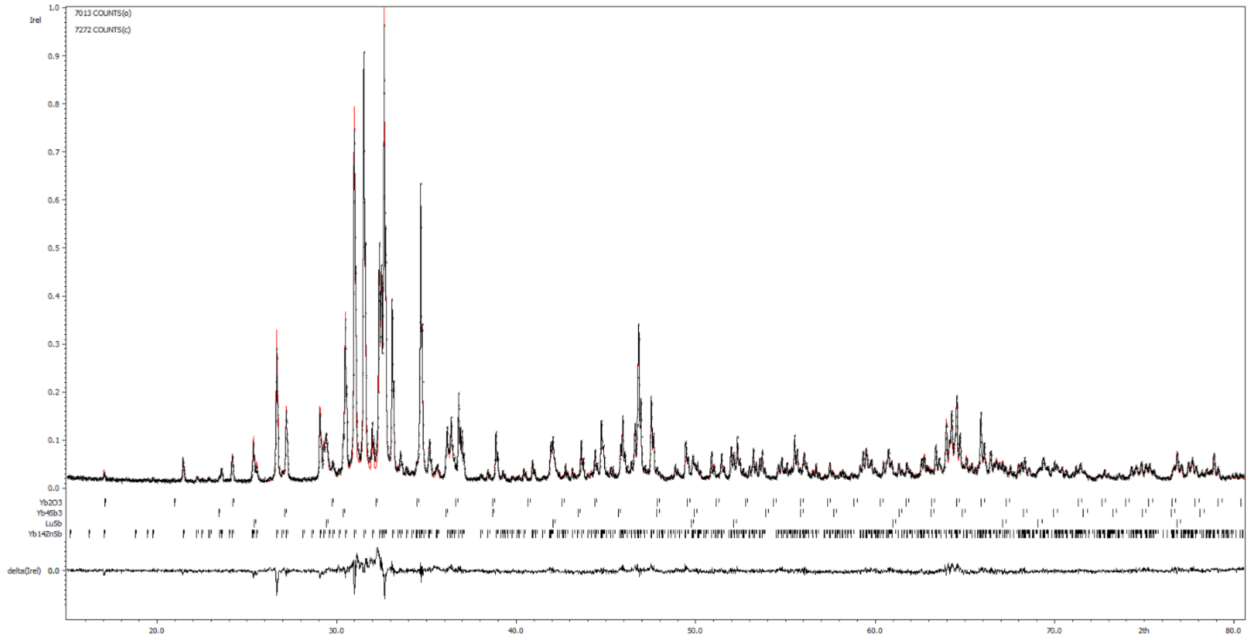

Figure S6: Rietveld refinement of the $Yb_{14-x}Lu_xZnSb_{11}$ $x = 0.6$ PXRD pattern.

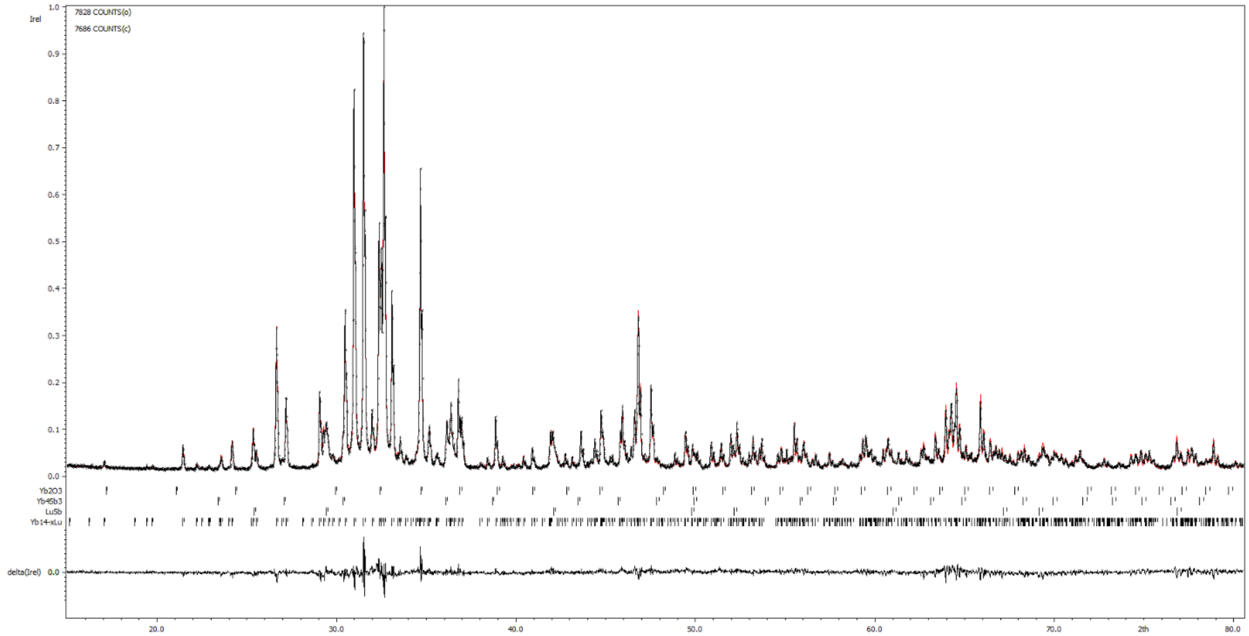

Figure S7: Rietveld refinement of the $Yb_{14-x}Lu_xZnSb_{11}$ $x = 0.7$ PXRD pattern.

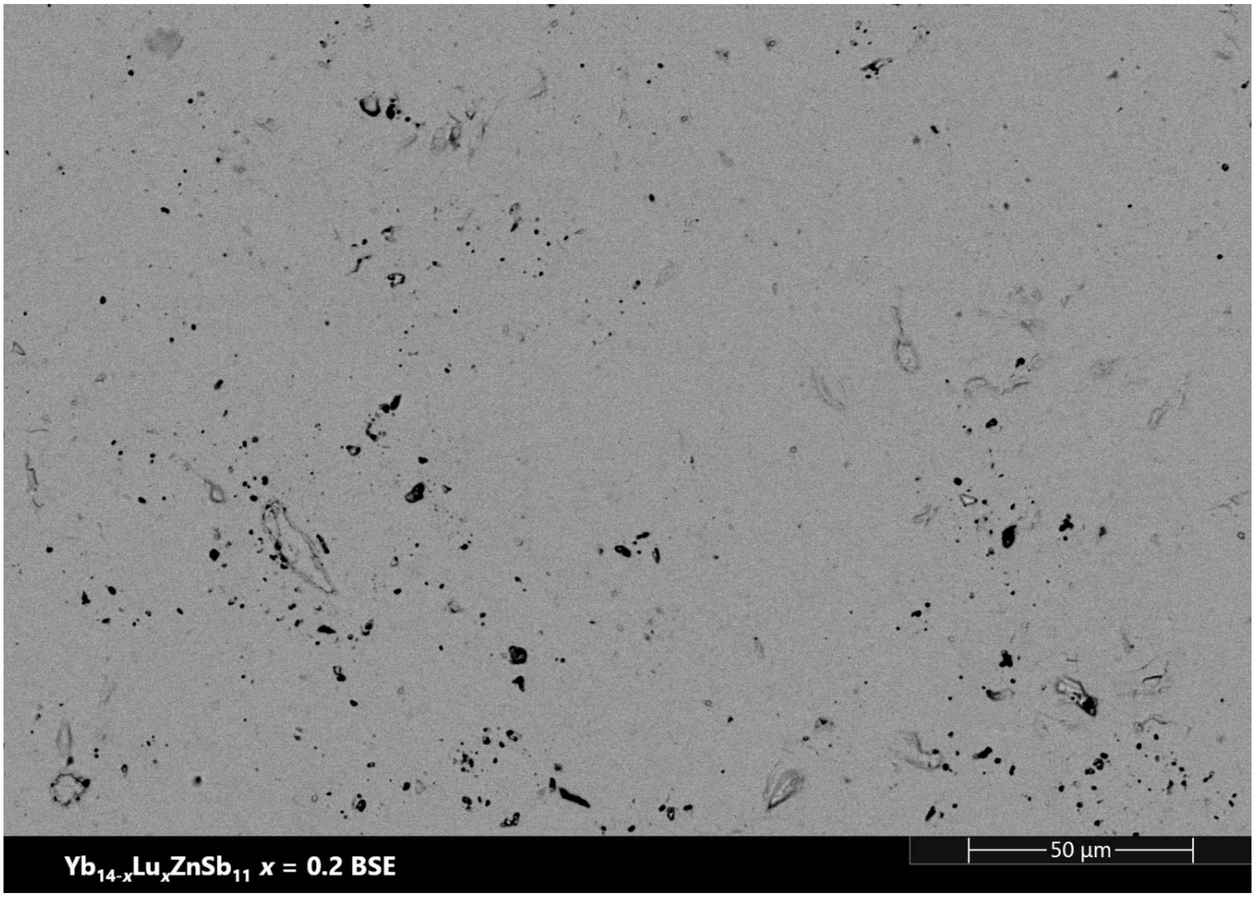

Figure S8. Backscattered electron micrograph of $Yb_{14-x}Lu_xZnSb_{11}$, x = 0.2.

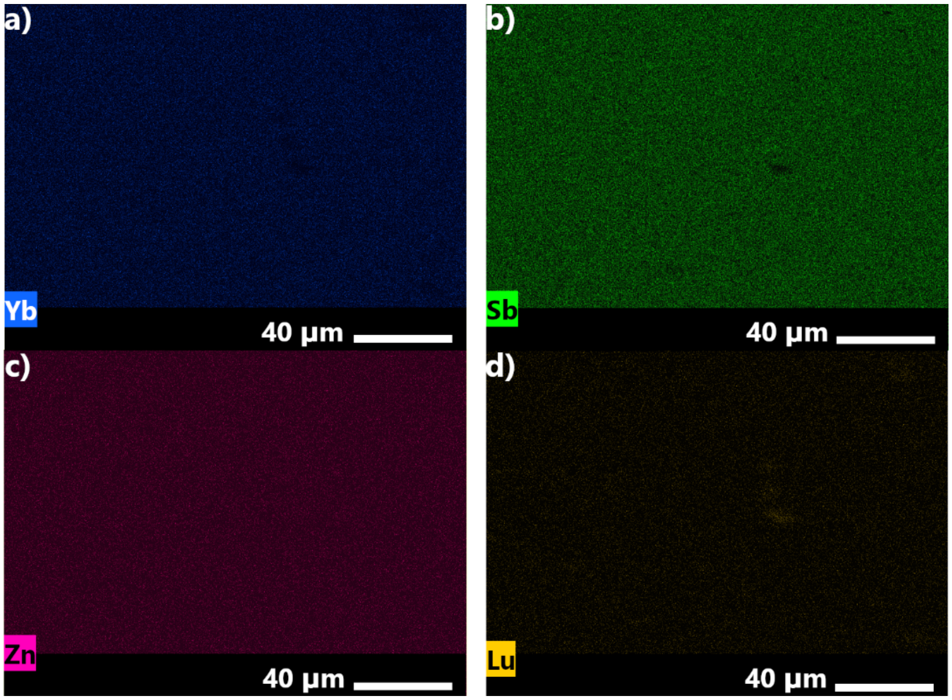

Figure S9. Elemental maps of a) Yb, b) Sb, c) Zn, and d) Lu from energy dispersive spectroscopy of $x = 0.2$. The element is indicated in the bottom left with a 40 µm scale bar on the right.

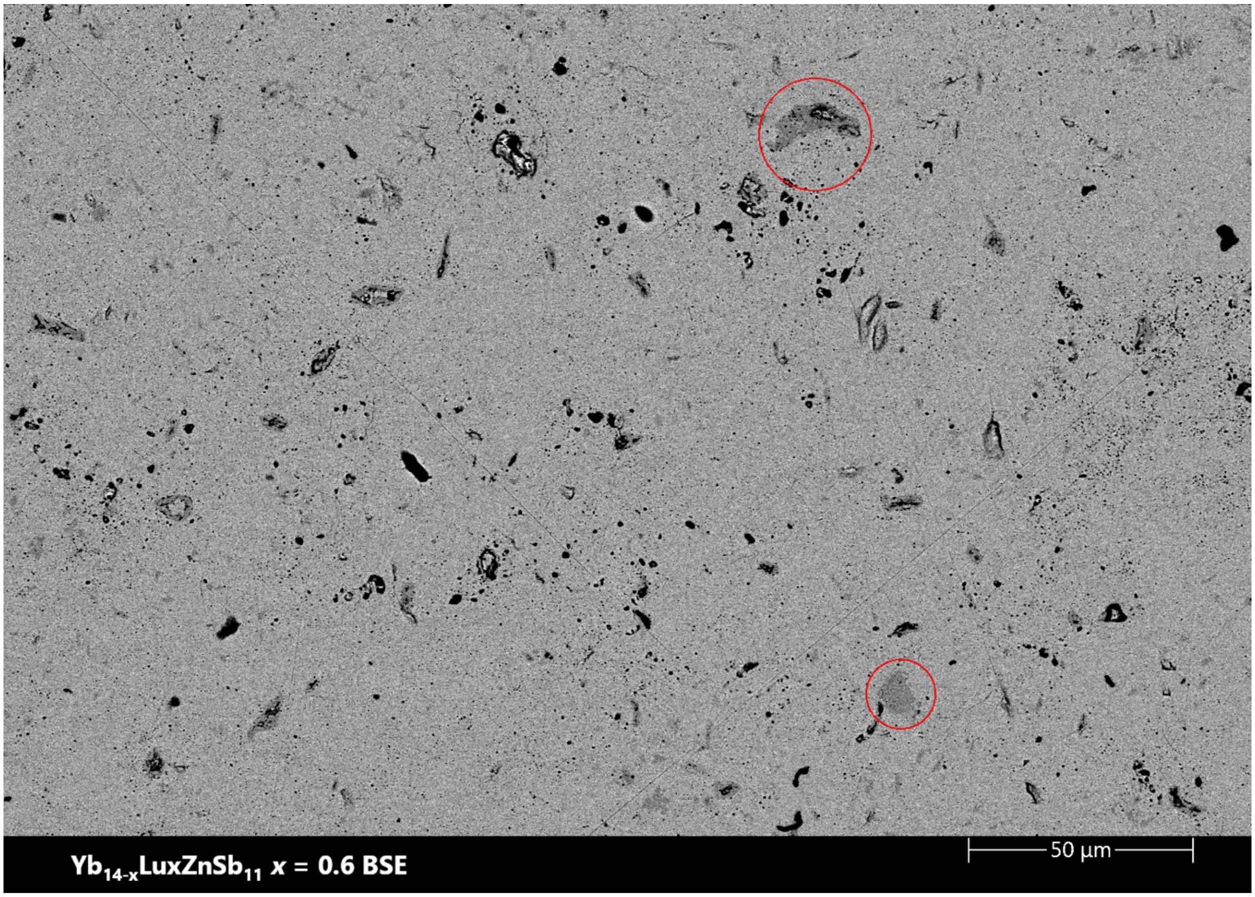

Figure S10. Backscattered electron micrograph of $Yb_{14-x}Lu_xZnSb_{11}$, x = 0.6. Corresponding regions of deficient Yb and excess Lu are circled in red. A 50 μm scale bar is indicated on the bottom right.

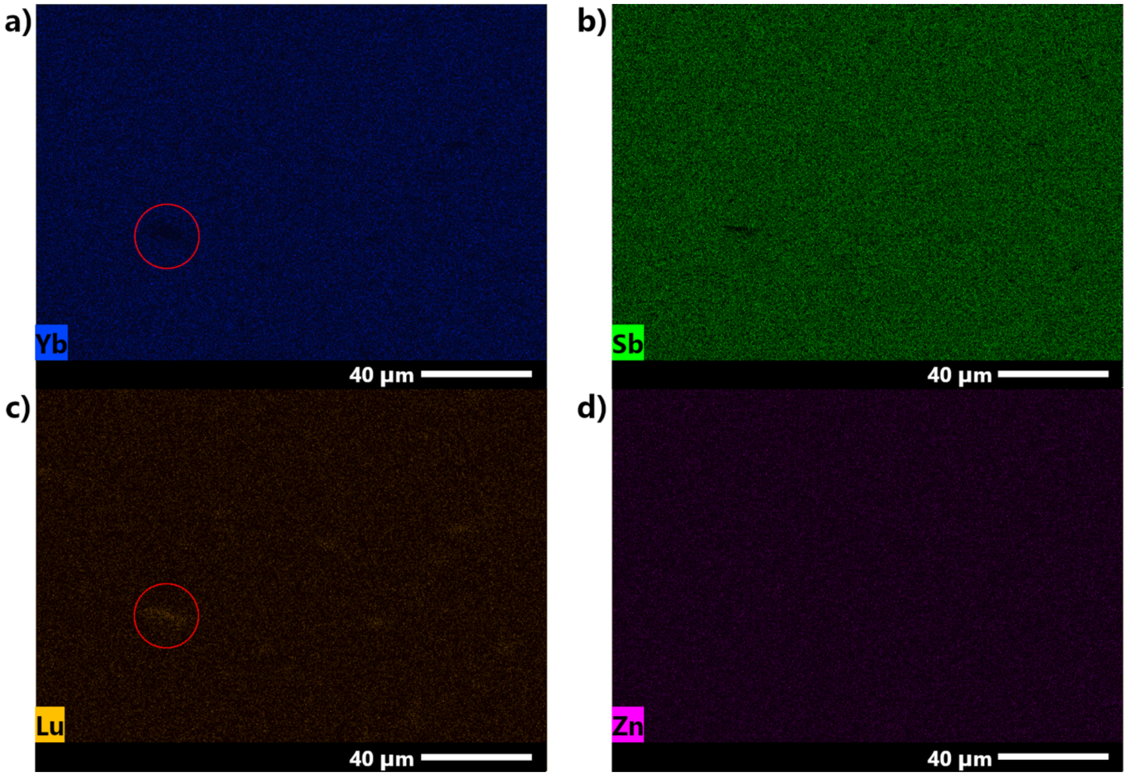

Figure S11. Elemental maps of a) Yb, b) Sb, c) Lu, and d) Zn from energy dispersive spectroscopy for $Yb_{14-x}Lu_xZnSb_{11}$, $x = 0.6$. Corresponding regions of deficient Yb and excess Lu are circled in red. The element is indicated in the bottom left with a 40 μm scale bar on the right.

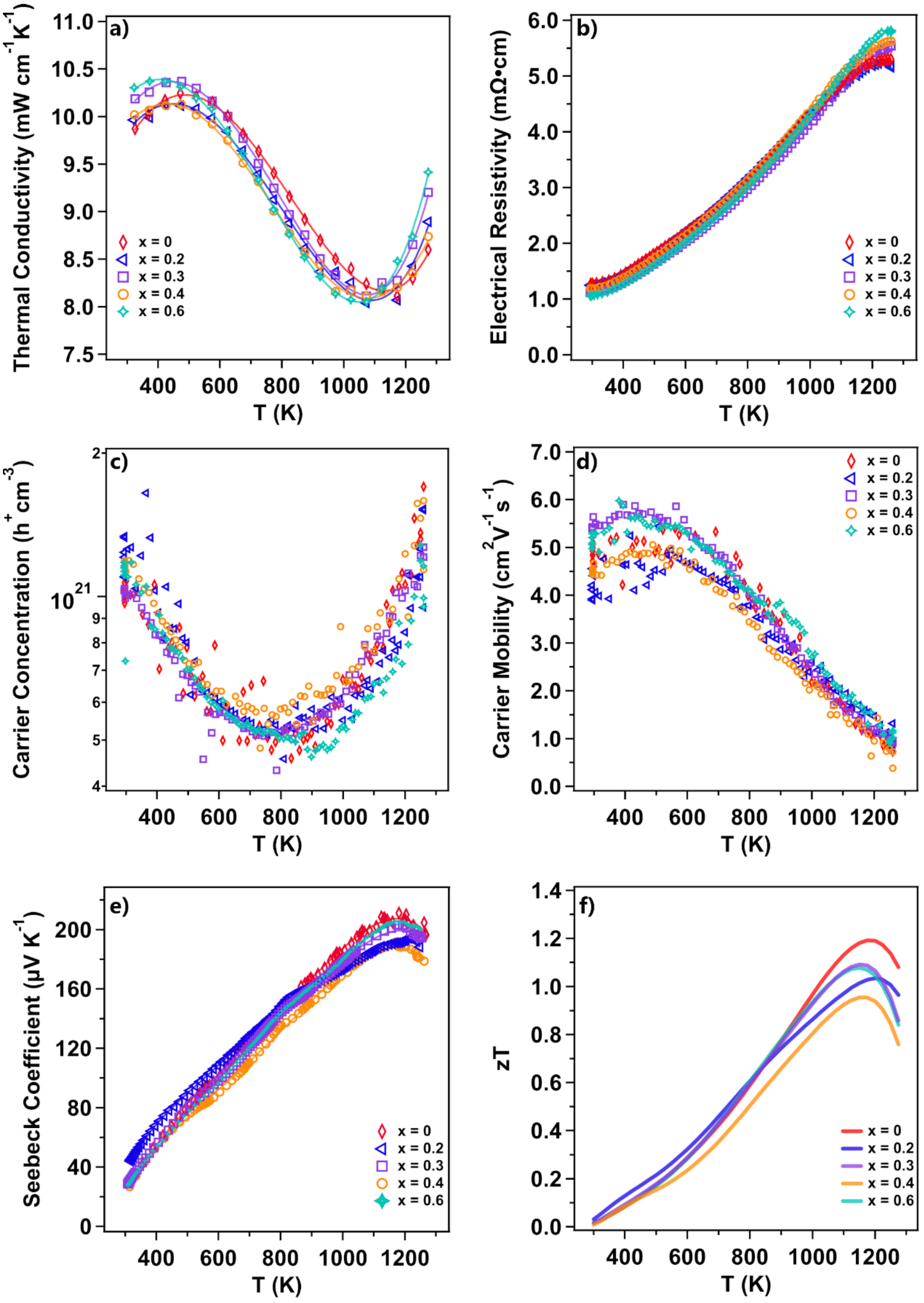

Figure S12. The a) thermal conductivity, b) electrical resistivity, c) Hall carrier concentration, d) carrier mobility, e) Seebeck coefficient, and f) *zT* of the $Yb_{14-x}Lu_xZnSb_{11}$ series with $x = 0.6$ included shown in teal.

Table S1. Room Temperature Hall Mobility and Carrier Concentration Values‡ for $Yb_{14-x}Lu_xZnSb_{11}$

$Yb_{14-x}Lu_xZnSb_{11}$	RT Mobility ($cm^2 V^{-1} s^{-1}$)	RT Carrier Concentration (cm^{-3})
$x = 0$	4.82	$1.03x10^{21}$
$x = 0.2$	4.17	$1.23 x10^{21}$
$x = 0.3$	5.45	$1.02 x10^{21}$
$x = 0.4$	4.52	$1.17 x10^{21}$
$x = 0.6$	5.16	$1.14 x10^{21}$

‡Values presented are the averages of data collected between 295 – 305 K.

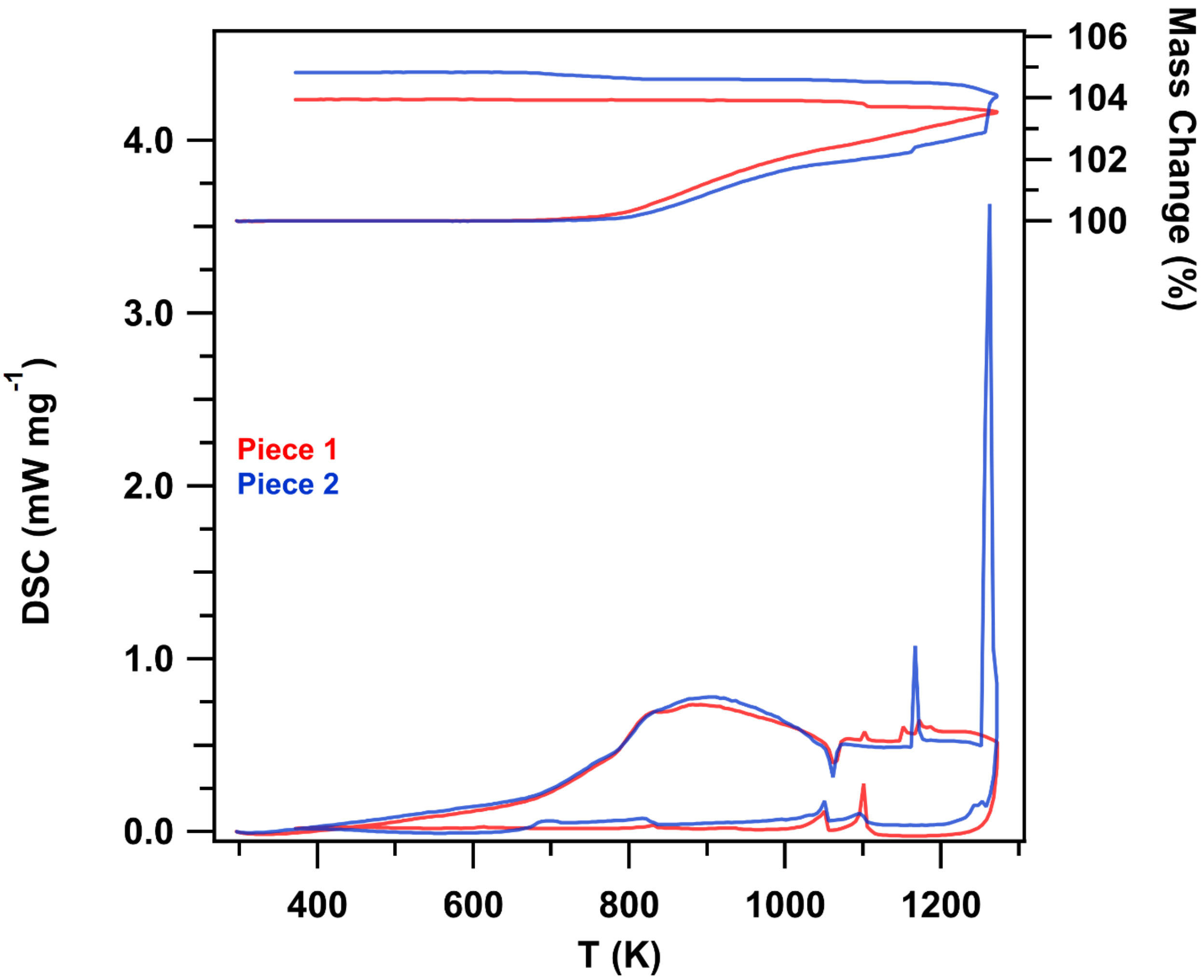

Figure S13. The complete TG trace (top, right axis) and DSC (bottom, left axis) for two pieces of $Yb_{14-x}Lu_xZnSb_{11}$ ($x = 0.3$) from room temperature to 1173 K under a 50 mL/ min flow of dry air.
